# Chloride and Acetonitrile Ruthenium(IV) Complexes: Crystal Architecture, Chemical Characterization, Antibiofilm Activity, and Bioavailability in Biological Systems

**DOI:** 10.3390/molecules30030564

**Published:** 2025-01-26

**Authors:** Agnieszka Jabłońska-Wawrzycka, Patrycja Rogala, Grzegorz Czerwonka, Maciej Hodorowicz, Justyna Kalinowska-Tłuścik, Marta Karpiel

**Affiliations:** 1 Institute of Chemistry, Jan Kochanowski University, 7 Uniwersytecka Str., 25-406 Kielce, Poland; 2 Institute of Biology, Jan Kochanowski University, 7 Uniwersytecka Str., 25-406 Kielce, Poland; gczerwonka@ujk.edu.pl; 3 Faculty of Chemistry, Jagiellonian University, 2 Gronostajowa Str., 30-387 Cracow, Poland; hodorowm@chemia.uj.edu.pl (M.H.); kalinows@chemia.uj.edu.pl (J.K.-T.); marta.karpiel@doctoral.uj.edu.pl (M.K.); 4 Doctoral School of Exact and Natural Sciences, Jagiellonian University, 11 Lojasiewicza Str., 30-348 Cracow, Poland

**Keywords:** ruthenium(IV) complex, molecular structure, biofilm, molecular docking studies

## Abstract

Due to the emergence of drug resistance, many antimicrobial medications are becoming less effective, complicating the treatment of infections. Therefore, it is crucial to develop new active agents. This article aims to explore the ruthenium(IV) complexes with the following formulas: (Hdma)_2_(HL)_2_[Ru^IV^Cl_6_]·2Cl·2H_2_O (**1**), where Hdma is protonated dimethylamine and L is 2-hydroxymethylbenzimidazole, and [Ru^IV^Cl_4_(AN)_2_]·H_2_O (**2**), where AN is acetonitrile. This paper delves into the physicochemical characteristics and crystal structures of these complexes, employing various techniques such as spectroscopy (IR, UV–Vis), electrochemistry (CV, DPV), and X-ray crystallography. Hirshfeld surface analysis was also performed to visualize intermolecular interactions. Furthermore, the potential antibiofilm activity of the complexes against *Pseudomonas aeruginosa* PAO1 was investigated and the effect of the compounds on the production of pyoverdine, one of the virulence factors of the *Pseudomonas* strain, was assessed. The results show that particularly complex **1** reduces biofilm formation and pyoverdine production. Additionally, the bioavailability of these complexes in biological systems (by fluorescence quenching of human serum albumin (HSA) and molecular docking studies) is discussed, assessing how their chemical properties influence their interactions with biological molecules and their potential therapeutic applications.

## 1. Introduction

Antibiotic resistance in bacteria presents a significant public health challenge that complicates the treatment of infections, contributes to increased mortality rates, and escalates healthcare costs. Exploring novel strategies to combat infections, including the use of metal complexes, may offer viable alternatives to traditional antibiotic therapies [1,2,3,4]. In recent years, chloride complexes of transition metals have gained importance due to their ability to adopt a diverse range of coordination geometries and oxidation states [5,6,7,8,9]. These complexes are also attractive because of their interesting chemical properties as hybrid materials and catalysts [10,11,12,13,14,15]. Among metal complexes, ruthenium complexes are particularly intriguing due to their unique properties [16] and potential applications as therapeutic agents [17,18,19,20,21]. It is essential that these complexes exhibit the ability to interact with biomolecules, which may affect their antimicrobial activity. However, further research is crucial to comprehensively understand their mechanisms of action, evaluate their effectiveness, and assess potential risks.

Long-term research on the preparation of ruthenium (Ru) complexes has demonstrated that, in some instances, chloride complexes form instead of chelating complexes [22,23]. It has been observed that in the presence of acetonitrile (AN), these complexes undergo substitution by solvent molecules [23]. The mechanism of this reaction, previously studied using cyclic voltammetry (CV), was elucidated earlier [22]. Acetonitrile is recognized as an exceptionally effective solvent in the field of coordination chemistry. Its polar characteristics enhance the solubility of salts derived from complex ions and various polar transition metal complexes. The ability of acetonitrile to function as a donor through the nitrile nitrogen contributes to the stabilization of low-coordinate species in solution, thereby facilitating their crystallization as nitrile adducts. When employed as a ligand, acetonitrile, coordinated via its nitrogen atom, exhibits sufficient reactivity to be readily substituted by alternative donor ligands, which accounts for its frequent use in transition metal precursor complexes. As a result, both stoichiometric and catalytic reactions conducted in acetonitrile often leverage this neutral nitrogen donor as a non-reactive placeholder ligand.

In several other cases, we have also synthesized chlorido-acetonitrile complexes in which a protonated organic ligand is present within the crystalline network. We discovered that an appropriately applied volumetric ratio of solvents (ethanol, EtOH, and acetonitrile, AN) facilitates the formation of chelating complexes substituted with an organic ligand, particularly those involving N,N-donor ligands. Unfortunately, this approach was not effective for N,O-donor ligands (such as alcohols and acids), which either underwent redox reactions or required the selection of an appropriate counterion. We hypothesize that the presence of ions [RuCl_6_]^3−^ and [RuCl_6_]^4−^ in the mother solution plays a significant role in these equilibria. Therefore, in this manuscript, we investigate the structure and properties of the complex [RuCl_4_(AN)_2_], which is likely a transitional stage in the synthesis of Ru complexes with chelating ligands. Several reports in the literature describe the structure and properties of Ru chlorocomplexes, including those that incorporate molecules of acetonitrile. For example, Ru(CH_3_CN)_2_Cl_4_, as described by Schumann, has been synthesized and studied in relation to its catalytic properties using electrochemical methods [15]. However, Schumann’s synthesis was based on an electrochemical method. Our synthesis method relies on the utilization of a mother solution and is competitive with the approach employed by Schumann. Its advantages include reduced time consumption and lower costs while maintaining a higher yield of synthesis. Therefore, in this work, we present a comprehensive approach to the ruthenium(IV) complex with acetonitrile, including an investigation of its magnetic behavior and spectroscopic properties. Furthermore, the novelty of our approach is demonstrated through a series of studies aimed at assessing its biological activity. To the best of our knowledge, the biological activity of the Ru complex with acetonitrile has not been previously described in the literature. Among the chloride complexes, we observed that the solvent used in the synthesis can become an integral part of the crystalline structure. Our previous project focused on biological applications, particularly the successful ability of the chloride ruthenium complex to inhibit biofilm formation by generating oxidative stress in bacterial cells [22] and disrupting the adhesion process in *Pseudomonas aeruginosa* PAO1 [24]. This finding underscores the therapeutic potential of these ruthenium-based compounds in combating resistant bacterial strains. Consequently, we examined the influence of the solvents employed (EtOH and dimethylamine, dma, in this work) on the chemical and biological properties of the complexes.

In this article, two ruthenium complexes in the +IV oxidation state were synthesized. The isolated compounds underwent spectroscopic characterization (FT-IR, UV–Vis), magnetic studies, and electrochemical analyses (CV, DPV). Their crystal structures (SC-XRD) were also determined and the Hirshfeld surface was calculated. Additionally, the impact of the Ru complexes on selected virulence factors was assessed. Given that the potential activity of these complexes is related to their affinity for binding with the blood transport protein, human serum albumin (HSA), understanding the interactions between HSA and the Ru complexes synthesized in this study is crucial for elucidating how these complexes are transported, distributed, and their bioavailability within the biological system. The molecular interactions of HSA with complexes **1** and **2**, as well as the impact of these complexes on the structure of HSA, were investigated using spectroscopic studies (fluorescence quenching) and molecular docking.

## 2. Results and Discussion

### 2.1. Preparation of Complexes and Their Structural Characterization by FT-IR and UV–Vis Spectroscopy and Magnetic Studies

**Figure 1:**
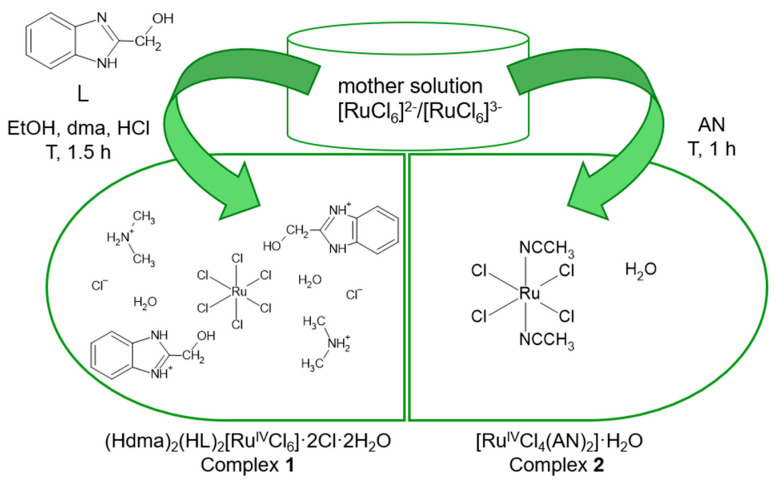
Synthesis of complexes **1** and **2**.

Considering that the chloride complexes of ruthenium previously obtained by our team exhibited significant antimicrobial activity [22], we decided to synthesize additional compounds of this type. The two chloride-ruthenium complexes presented in this paper were prepared by reacting a 0.1 M mother solution, which contains [RuCl_6_]^2−^/[RuCl_6_]^3−^ ions, with 2-hydroxymethylbenzimidazole (L) in a 1:2 molar ratio (complex **1**), and with acetonitrile (complex **2**) (Figure 1). In comparison with our previous study, the modification of complex **1** with 2-hydroxymethylbenzimidazole involved the use of dimethylamine as a solvent. Consequently, we obtained (Hdma)_2_(HL)_2_[Ru^IV^Cl_6_]·2Cl·2H_2_O, where hexachlororuthenate(IV) is balanced by the protonated ligand and protonated dimethylamine. The donor atoms of the organic ligand and solvent were blocked (via protonation) in the presence of concentrated hydrochloric acid and under low-temperature crystallization conditions. The synthesis of the second complex involved the combination of ruthenium(IV) ions with acetonitrile to create a compound that could serve as a precursor capable of ligand exchange. The result of the synthesis was complex **2**, which has the composition [Ru^IV^Cl_4_(AN)_2_]·H_2_O.

The composition and properties of these compounds were determined based on elemental analysis, spectroscopic methods (IR, UV–Vis), electrospray ionization mass spectrometry (ESI-MS), and magnetic and electrochemical studies (CV, DPV). Complexes **1** and **2** were also characterized by single-crystal X-ray diffraction analysis (SC-XRD) and Hirshfeld surface analysis (HS). Additionally, for comparative analysis, the protonated form of the ligand (which was prepared in the solid state and whose chemical composition was confirmed using spectroscopic methods; the spectroscopic description of this compound is presented in [22]) was also tested in some experiments. Complex **1** exhibits bands at approximately 3500 cm^−1^ and 3240 cm^−1^, which can be assigned to the stretching vibrations of O–H groups from water molecules and the alcohol group in the ligand, respectively. Additionally, the IR spectrum of this compound demonstrates strong, sharp bands at 3206 cm^−1^ and 3142 cm^−1^, corresponding to the protonated amine and the protonated nitrogen in the aromatic ring. Bands in the region of 1650–1420 cm^−1^, attributed to the C=C and C=N stretching modes of the benzimidazole ring, are also observed. The shift of these vibrations to higher energies, compared with the frequencies of the free ligand, suggests that the nitrogen atom of the ligand is not involved in coordination with the ruthenium ion. The protonated form of benzimidazole also appears in the complex we obtained earlier: (H_3_O)_2_(HL)_2_[RuCl_6_]·2Cl·2EtOH [22]. In the IR spectrum of complex **2**, the band observed in the range of 3600–3450 cm^−1^ confirms the presence of valence vibrations associated with the O–H group. This range is characteristic of a water molecule that is integrated within the crystal lattice and suggests the formation of hydrogen bonds with differing strengths. The presence of acetonitrile in complex **2** is supported by the occurrence of bands at 2981 and 2922 cm^−1^ (C–H stretching vibrations of the methyl group in the acetonitrile molecule) and at 2295 cm^−1^ (the valence vibrations of the C≡N group). A similar value for the CN vibration was observed in other ruthenium-acetonitrile complexes [25]. The shift towards higher wavenumbers of the band responsible for the ν_C≡N_ vibrations, compared to the vibration of free acetonitrile (2250 cm^−1^) [26], indicates the involvement of the nitrogen atom in the formation of a coordination bond with the central ion. The selected IR assignments for complexes **1** and **2** are given in Table S1 (ESI).

The electronic absorption spectra of 2-hydroxymethylbenzimidazole and complexes **1** and **2** were recorded in the range of 200–800 nm (Figure S1) in two solvents with distinct chemical properties: water (highly polar and strongly acidic) and dimethyl sulfoxide (polar aprotic and moderately basic). The organic ligand displayed bands in both solvents at 210–280 nm due to π → π* transitions, while complex **1** exhibited corresponding bands at 220–275 nm. In the UV–Vis spectra of complex **2**, four bands (in water) and three bands (in DMSO) in the range of 200–300 nm associated with π → π* transitions in the acetonitrile molecules were observed. Band maxima related to ligand-to-metal charge transfer (LMCT) transitions from the chloride ion to the metal center were observed in the range of 325–420 nm in the tested compounds. In complex **1**, these bands were detected at 325 and 360 nm (in water), and at 418 nm (in DMSO). For complex **2**, the bands were observed at 379 and 392 nm (in water), and at 400 and 415 nm (in DMSO). For both compounds, a bathochromic shift of the LMCT bands was observed as the solvent polarity decreased (Figure S2, Table 1). This can be attributed to the polar nature of water, which is a protic solvent and causes a different type of interaction with the compounds compared to DMSO, an aprotic solvent. Additionally, the absorption spectra revealed the presence of low-intensity bands at 491 nm (in water) and 498 nm (in DMSO) for complex **1**, and at 454 nm (in water) and 477 nm (in DMSO) for complex **2**, which were attributed to *d–d* transitions. According to the Tanabe–Sugano diagram, the *d–d* transitions have been assigned to the ^3^T_1g_→^3^E_g_ transitions for the low-spin Ru(IV) complex (t_2g_^4^e_g_^0^ configuration) in an O_h_ environment. The UV–Vis method was also used to evaluate the stability of the obtained ruthenium complexes. Spectra were recorded for the compounds in two solvents: water and DMSO, at both 0 h and 24 h to assess their stability over the period designated for biological studies. The results indicated that complexes **1** and **2** are stable in aqueous solution over the 24–hour period, as evidenced by the absence of shifts in the relevant bands and only slight changes in the intensity of the absorption maxima. Similar observations were made for the complexes dissolved in DMSO. The UV–Vis spectra of Ru complexes in the tested solvents, recorded immediately after preparation and after 24 h, are included in the electronic supplementary information (Figure S3a–d).

The electrospray ionization technique in positive mode was used for mass spectrometric studies of the chloride ruthenium complexes. The experimental *m*/*z* values, as well as the isotopic distribution patterns, are in good agreement with the calculated values. The spectra confirm the compositions of the complexes as determined by other analytical methods.

Magnetic susceptibility measurements of complexes **1** and **2** using the Gouy method (at room temperature) showed the paramagnetic nature of the compounds. The values of μ_eff_ obtained for the complexes (2.49 μB and 2.32 μB) correspond to two unpaired electrons (S = 1) for the Ru(IV) ions in an octahedral environment.

### 2.2. Description of the Molecular and Crystal Structure for Complexes ***1*** and ***2***

**Figure 2:**
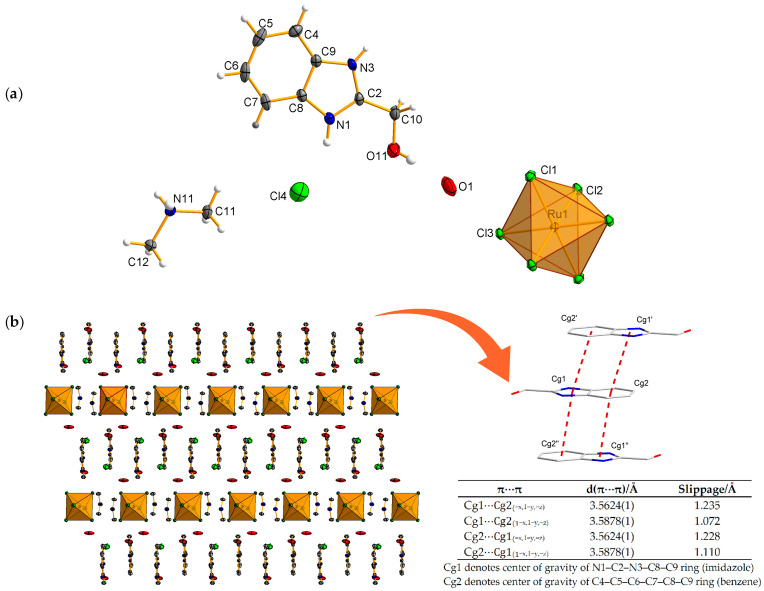
Molecular and crystal structure of compound **1** with a numbering scheme. All atoms shown are represented on thermal ellipsoids with a probability of 30% (**a**). Crystal packing with marked intermolecular π···π stacking interactions between aromatic rings belonging to the 2-(hydroxymethyl)-1*H*-benzimidazol-3-ium ion (**b**).

Single crystal X-ray analysis revealed that the asymmetric part of the unit cell of complex **1** contains ruthenium(IV) ions placed at the inversion symmetry center (with occupancy 0.5), together with three coordinating chloride anions in the general positions. Additionally, the composition is completed with one molecule of protonated 2-(hydroxymethyl)-1*H*-benzimidazol-3-ium ion, one chloride counter-anion, a water molecule, and one protonated dimethylamine (Hdma) molecule (Figure 2a). The composition of complex **1** can be best expressed by the formula (Hdma)_2_(HL)_2_[Ru^IV^Cl_6_]·2Cl·2H_2_O. The geometry of the coordination environment of the Ru(IV) atom is octahedral, with six chloride ions positioned at a relatively long but nearly symmetric distance of about 2.374(5) Å (Table 2). Key bond distances and angles are detailed in Table 2. The Ru–Cl bond is slightly longer than that reported by Liu et al. [13] and Sharutin [27], but it is similar to the value for other complexes examined by our team [23].

The crystal structure of complex **1** features alternating layers that are oriented parallel to *b* axis, comprising discrete centrosymmetric [Ru^IV^Cl_6_]^2−^ octahedra and 2-(hydroxymethyl)-1*H*-benzimidazol-3-ium ions (Figure 2b). A supramolecular analysis of the crystal structure indicates that the complex molecules are interconnected through hydrogen bonds of the N–H⋯Cl and C–H⋯Cl types, as well as intermolecular π⋯π stacking interactions between the aromatic rings of the 2-(hydroxymethyl)-1*H*-benzimidazol-3-ium ions (Table S2 and Figure 2b). An interesting feature of the structure of complex **1** is the presence of the Hdma molecule, which forms as many as six hydrogen bonds: five of the N–H⋯Cl type and one of the C–H⋯Cl type, with the coordinating Ru(IV) chloride anions, and acts as a molecular “clip” in the structure. The same type of intermolecular interactions is observed in the complex with ethanol [22].

**Figure 3:**
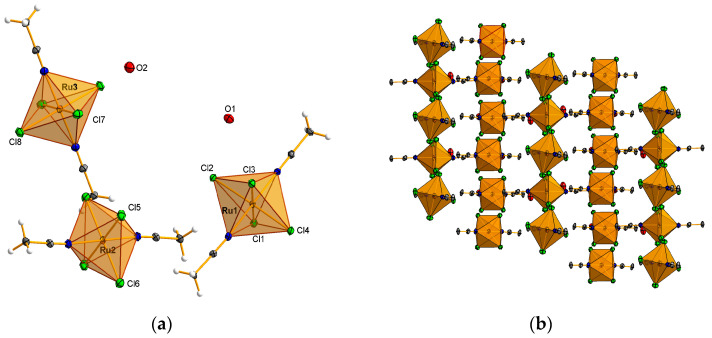
Molecular structure of compound **2** with a numbering scheme. All atoms shown are represented on thermal ellipsoids with a probability of 30% (**a**). The spatial arrangement of RuC_l4_(AN)_2_ units in the crystal of compound **2** displaying layered, alternating arrangement with a clear distinction between two layers (**b**).

X-ray analysis of the crystals of compound **2** reveals that the asymmetric part of the unit cell contains discrete, well-defined three RuCl_4_(AN)_2_ units, with two of them based on Ru2 and Ru3 atoms occupying special positions (inversion center) (Figure 3a). For all three ruthenium atoms, the coordination environment, comprising the four chlorine atoms in the octahedral base and the two nitrogen atoms from the acetonitrile molecules in axial positions, adopts a flattened octahedral geometry. This distortion is manifested in the interatomic distances and valence angles in the polyhedron (Table 3). For example, the chlorine atoms are located at an average distance from the Ru atom of 2.348(6) Å, with an average Cl–Ru–Cl angle of 90.0(5)° within the margin of error (Table 3), while the nitrogen atoms remain at an average distance of 2.03 Å. The average Ru–N bond lengths are longer than those found in other ruthenium chloride nitrile complexes [15,25], but they are similar to those reported in [28]. The nitrogen atoms of the acetonitrile groups are slightly tilted from the N–Ru–N axis, as evidenced by an average angle of 179(2)°. This slight deviation from linear geometry also applies to the acetonitrile molecules and takes values in the range of 177.9(1)–179.3(1)°.

The spatial arrangement of RuCl_4_(AN)_2_ units in the crystal of compound **2** is shown in Figure 3b. Their layered, alternating arrangement is evident, with a clear distinction between two layers, the first one composed of molecules based on Ru2 and Ru3 atoms (Ru2/Ru3) and the second one on ruthenium atoms Ru1. Numerous hydrogen interactions of the C–H⋯Cl type play a fundamental role in stabilizing the crystal structure of compound **2** (Table S2).

### 2.3. Hirshfeld Surface Analysis (HS)

The intermolecular interactions discussed in the preceding section were confirmed through the analysis of Hirshfeld surfaces. The Hirshfeld surface and the two-dimensional (2D) fingerprint plot of the complexes were generated using the corresponding Crystallographic Information File (CIF) as an input. Subsequently, the quantification and decoding of inter-contact interactions in the crystal packing were visualized using the *d*_norm_ descriptor and the 2D fingerprint plot, respectively.

**Figure 4:**
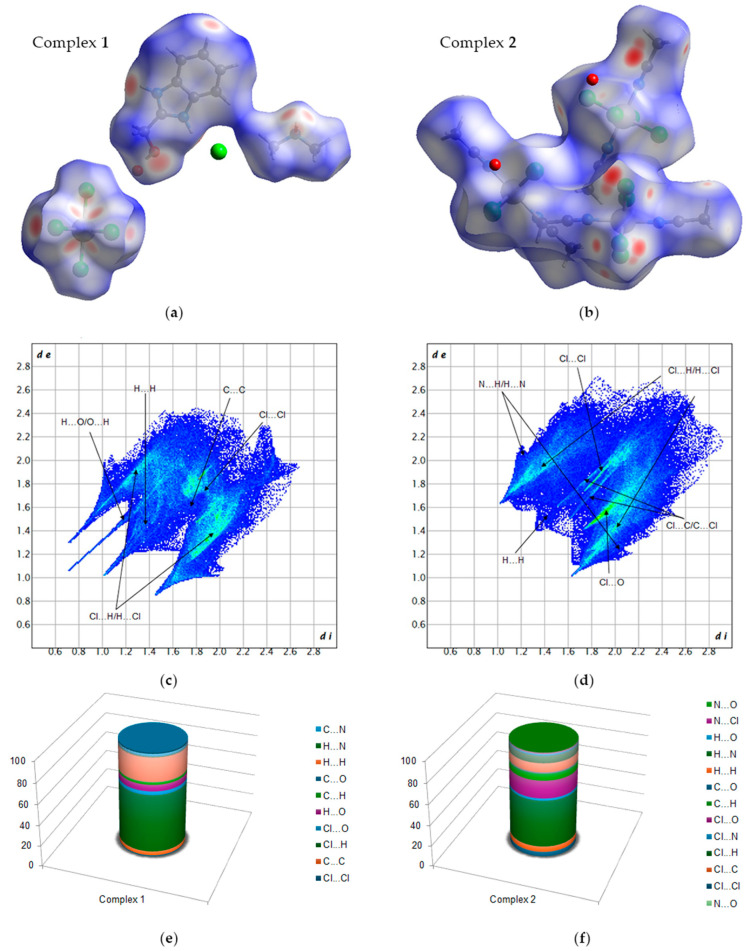
The Hirschfeld surfaces highlight the relevant *d*_norm_ surface patches associated with the specific contacts for ruthenium complexes (**a**,**b**). The 2D fingerprint plots of the all of intermolecular interactions for Ru complexes (**c**,**d**) with percentage of interaction. Relative percentage contributions of close contacts to the Hirshfeld surface in Ru complexes (**e**,**f**).

In complex **1**, the red spots observed on the Hirshfeld surface confirm the presence of non-covalent interactions, primarily comprising N–H⋯Cl, C–H⋯Cl, and O–H⋯O hydrogen bonds (Figure 4a), as described in Section 3.5.

The analysis of the relative percentage contributions of close contacts to the Hirshfeld surface (Figure 4e) indicates that intermolecular Cl⋯H/H⋯Cl contacts constitute the most abundant interactions, accounting for 55.9%. These contacts are presented as two prominent outer spikes on the fingerprint plots, indicative of strong hydrogen bond interactions (Figure 4c). The second most frequent interactions involve weak intermolecular H⋯H contacts, accounting for 22.6% of the total (Figure 4e). These contacts manifest as the largest region on the fingerprint plots (Figure 4c). Smaller contributions to the Hirshfeld surface area are associated with H⋯O/O⋯H contacts, which account for 6.2% and are represented by a very sharp internal spike in the fingerprint plot (Figure 4c,e). These contacts are linked to the presence of O–H⋯O type hydrogen bonds between the organic ligand and the water molecule. Furthermore, we observed that for *d*_i_ + *d*_e_ > 2.4 Å, the interaction between carbon atoms (3.9%) is also present. This percentage is indicative of the π⋯π stacking interaction with the atomic arrangement. Cl⋯Cl contacts are noteworthy, as they appear on the fingerprint plot as relatively strong interactions, despite their contribution being only 0.9%. Figure 4e demonstrates that for *d*_i_ + *d*_e_ ≥ 3.5 Å, the interaction between chlorine atoms (Cl⋯Cl) can be considered to range from relatively strong to moderate.

Hirshfeld surface (HS) analysis and 2D fingerprint plots revealed various intermolecular interactions in the crystal structure of complex **2** (Figure 4b). Red spots on the Hirshfeld surface indicate the presence of reciprocal contacts involving Cl⋯H, Cl⋯O, Cl⋯C, and Cl⋯Cl. The 2D fingerprint plot demonstrates that the crystal packing is predominantly influenced by Cl⋯H/H⋯Cl contacts, which account for 45.4% of the total interactions (Figure 4f). These contacts are depicted in the 2D plot as two symmetric external spikes, indicating the presence of numerous C-H⋯Cl type hydrogen interactions (Figure 4d). A significant contribution to the Hirshfeld surface is also made by Cl⋯O/O⋯Cl contacts (Figure 4d,f), which account for 16.8%. These contacts are associated with interactions between coordinated chloride ions and water molecules. H⋯H contacts appear in the 2D fingerprint plots as widely scattered points of low density, reflecting the low hydrogen content in the molecule. Their contribution to the Hirshfeld surface is estimated to be 8.3% (Figure 4f). The contributions of H⋯N/N⋯H contacts in the crystal are represented as characteristic wings, accounting for 6.9% of the surface contribution (Figure 4d,f). Additional smaller contributions from other intermolecular contacts on the Hirshfeld surface include Cl⋯C/C⋯Cl (5.4%) and Cl⋯Cl (4.5%).

### 2.4. Electrochemical Studies

**Figure 5:**
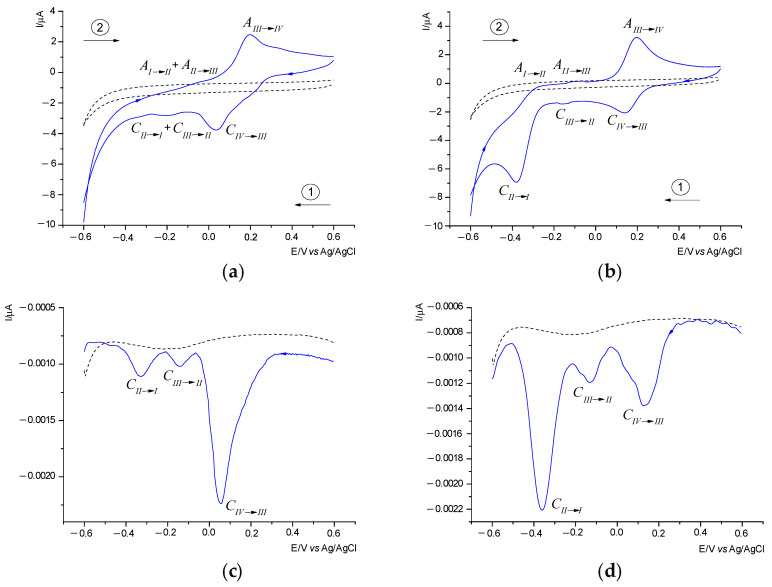
CV (**a**,**c**) and DPV (**b**,**d**) curves recorded in in a mixture of acetonitrile and ethanol, with 0.1 M TBAPF_6_ and 1 mM ruthenium complexes **1** and **2** (—). Line (---) indicates ligand or supporting electrolyte. CV conditions: GCE, Ø = 2 mm, scan rate 50 mVs^−1^, T = 25 °C; DPV conditions: CF, Ø = 33 µm, pulse amplitude 20 mV, pulse width 80 ms, scan rate 20 mV s^−1^.

The redox properties of the ruthenium(IV) complexes were investigated using cyclic voltammetry (CV) and differential pulse voltammetry (DPV) in a mixture of acetonitrile and ethanol, with TBAPF_6_ serving as the supporting electrolyte. The voltammograms for the complexes were recorded using a glassy carbon (GC) working electrode for CV and a carbon fiber (CF) working electrode for DPV, utilizing an Ag/AgCl reference electrode. Figure 5 presents the representative voltammograms of complexes **1** and **2** within the potential range of −0.6 to 0.6 V at a scan rate of 50 mV s^−1^. It is important to highlight that the ligand appears to lack electroactivity under the experimental conditions (indicated by the dashed line in Figure 5a,b).

Both Ru(IV) complexes displayed similar redox behavior. The cathodic peaks corresponding to the transition from Ru(IV) to Ru(III) were observed at potentials of 0.160 V (complex **1**) and 0.139 V (complex **2**), while the anodic peaks associated with the oxidation of Ru(III) to Ru(IV) appeared at potentials of 0.200 V and 0.196 V, respectively (Table 4). Broad signals detected at approximately −0.210 V and −0.055 V for complex **1** can be attributed to overlapping signals from the reduction of Ru(III) to Ru(II) and Ru(II) to Ru(I), as well as the oxidation of Ru(I) to Ru(II) and Ru(II) to Ru(III). The application of the DPV technique allowed for the distinction of closely spaced peaks on the reduction curve. In Figure 5b, two distinct signals are observed: one at −0.140 V corresponding to the C_III→II_ peak and another at −0.330 V related to the C_II→I_ peak. For complex **2**, weakly defined cathodic (~−0.150 V) and anodic (~−0.085 V) peaks were assigned to the Ru(III)↔Ru(II) redox couple (Figure 5c). The signals for the reduction of Ru(II) to Ru(I) and the oxidation of Ru(I) to Ru(II) occur at −0.380 V and −0.260 V, respectively (Table 4). An examination of the DPV curve (Figure 5d) also reveals three distinct peaks, corresponding to the reduction of ruthenium in the +IV (0.131 V), +III (−0.132 V), and +II (−0.360 V) oxidation states. To assess the reversibility of the redox couples and the number of electrons transferred, the CV diagnostic criteria, specifically Δ*E*_p_ = *E*_pa_ − *E*_pc_ (where *E*_pa_ and *E*_pc_ represent the potentials of the anodic and cathodic peaks, respectively), were utilized [29]. These criteria indicate the exchange of one electron in all recorded cases. Additionally, the calculated peak-to-peak separation (Δ*E*_p_ for V = 0.05 V·s^−1^) for the Ru(IV)/Ru(III) redox pair in complex **1**, as well as for the Ru(II)/Ru(I) redox pair in complex **2**, significantly exceeds the theoretical value of 0.058 V for a reversible one-electron redox couple [29], demonstrating the irreversible nature of these systems. Slight changes in the Δ*E*_p_ values with increasing scan rate confirm the *quasi*-reversible nature of the Ru(III)/Ru(II) redox peak in complex **2**. In contrast, the Δ*E*_p_ value of 0.057 V for the Ru(IV)/Ru(III) redox pair in complex **2** is similar to the theoretically expected value, indicating a reversible process. The reversibility of the Ru(III)/Ru(II) and Ru(II)/Ru(I) couples is difficult to assess using the criteria typically employed in cyclic voltammetry.

Both complexes were monitored using electrochemical methods over several days to assess the impact of the solvents employed on any changes in the composition of the compounds. For complex **2**, no changes were observed during this period. In contrast, complex **1** exhibited noticeable changes as early as the second day of measurements. Specifically, while three signals were detected on the differential pulse voltammetry curve on the first day, a fourth signal appeared on the second day. This new cathodic peak, designated C’_IV→III_, emerged at 0.157 V and was associated with the formation of a new ruthenium(IV) complex (Figure S4). Concurrently, the C_IV→III_ peak at 0.055 V, indicative of the RuCl_6_^2−^ complex, gradually diminished over time. This decrease correlated with an increase in the intensity of the C’_IV→III_ peak at 0.157 V (Figure S4). This observation suggests that the +IV oxidation state of ruthenium ions demonstrates greater stability within the acetonitrile coordination sphere compared to the chloride coordination environment. Consequently, we infer that the RuCl_6_^2−^ anion in complex **1** undergoes the substitution of two chloride anions for two acetonitrile molecules, resulting in a compound composition similar to that of complex **2**. A similar relationship was also observed in the previously described chloride complex of ruthenium(IV) [22].

### 2.5. Minimum Inhibitory Concentration and Biofilm Formation Assay

The bacteriostatic properties of the synthesized ruthenium complexes, along with the antibiotic streptomycin, were evaluated by determining their minimum inhibitory concentration (MIC) against *Escherichia coli* ATCC 8739, *Staphylococcus aureus* ATCC 6538P, and *Pseudomonas aeruginosa* PAO1. This assessment was conducted using a broth microdilution assay, and the results are summarized in Table S3. An inhibitory effect was observed exclusively with complex **1**, which effectively inhibited the growth of *E. coli* and *P. aeruginosa* at the highest tested concentration of 1 mM. Such a result is similar to the result we obtained earlier for the chloride ruthenium(IV) complex with 2-hydroxymethylbenzimidazole [22]. The other compounds evaluated did not show any activity against planktonic cells. Streptomycin as the control inhibited bacterial growth at concentrations of 73 μg/mL and below (≤0.125 mM).

**Figure 6:**
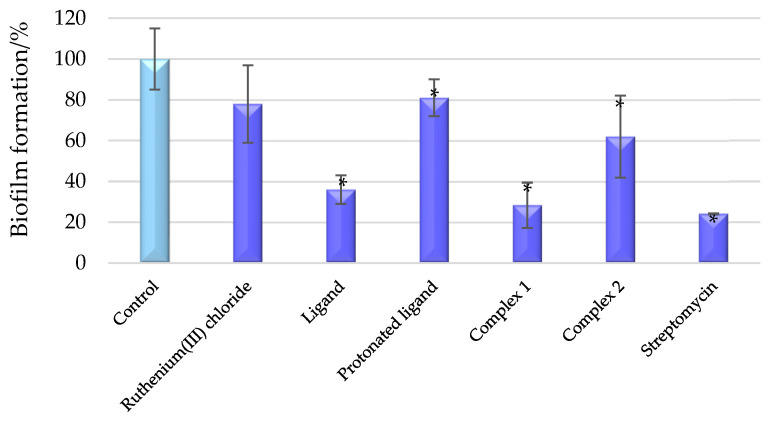
Biofilm formation of *P. aeruginosa* PAO1 in the presence of the tested compounds (concentrations of compounds—1 mM). The absorbance of the control group was taken as 100% biofilm formation. Results were deemed significant when compared to the control group (* *p* < 0.05). Data are presented as mean ± SD, *n* = 4.

While bacteria are unicellular organisms, they primarily exist in natural environments within biofilms. These single-celled organisms form biofilms because these structures can protect them from extreme temperatures, ultraviolet radiation, limited nutrients, antibiotics, and other stressors [30]. Consequently, biofilms become more difficult to eliminate using standard methods. To assess the ability of the obtained ruthenium complexes to inhibit biofilm formation by the *P. aeruginosa* PAO1 isolate, bacterial cells were treated with complex **1** and complex **2** at a concentration of 1 mM. For comparative analysis, ruthenium(III) chloride, ligand, and the protonated form of the ligand were also tested at the same concentration for their antibiofilm activity. The studies were conducted using crystal violet staining of the biofilm. The results of these experiments are illustrated in Figure 6. As depicted in Figure 6, the RuCl_3_, the protonated organic ligand, and complex **2** did not exhibit a significant inhibitory effect on *P. aeruginosa* PAO1 biofilm formation. In contrast, complex **1** was found to inhibit biofilm formation in the PAO1 strain by upwards of 70% at a concentration of 1 mM. It is worth mentioning that a previous study involving the same ligand and Ru(IV) ion demonstrated a similar level of inhibitory activity (78%) [22]. Furthermore, these values are comparable to those obtained for the antibiotic streptomycin. The results of the antibiofilm assay for complex **1** indicate that the effect of the [RuCl_6_]^2−^ anion is the most significant contributor to the activity of the compound, as only a minor antibiofilm effect is observed for the protonated ligand.

### 2.6. Regularity Between Electrochemical Parameters and Anti-Biofilm Activity

The importance of electrochemical techniques in the analysis of synthetic agents, particularly in relation to their biological properties (antibacterial, antifungal, antiparasitic, and anticancer activities), is continuously growing and cannot be overstated. Despite the complexity of the relationships in biomedical chemistry, certain correlations can be identified between biological and electrochemical parameters, which allow for the use of redox couples properties (electron transfer pathways—ET) to inhibit bacterial growth. The relationship between biological activity and the electrochemical parameters of chloride ruthenium(IV) complexes (studied by our team using cyclic voltammetry and differential pulse voltammetry) demonstrates a consistent trend. The correlation of biological activity (% biofilm inhibition) with the half-wave potentials of the Ru(IV)/Ru(III) couples shows a decreasing trend; specifically, as the half-wave potential increases, biological activity decreases. Furthermore, it was observed that chloride Ru(IV) complexes exhibited higher biological activity compared to chloride/acetonitrile complexes.

### 2.7. Pyoverdine Inhibition Assay

Pyoverdine plays a significant role in the virulence of *Pseudomonas* strains by influencing biofilm formation through various mechanisms. As a siderophore produced by several *Pseudomonas* species, including the human pathogen *Pseudomonas aeruginosa*, its primary function is to scavenge iron, an essential nutrient for bacterial growth. This iron acquisition not only supports bacterial survival in hostile environments but also enhances the pathogen’s ability to establish infections. In addition to its role in iron uptake, pyoverdine functions as a signaling molecule that stimulates the expression of various virulence factors, further increasing the pathogenicity of *Pseudomonas aeruginosa*. Moreover, pyoverdine affects biofilm formation and can modulate the host immune response, creating a protective environment that facilitates chronic infections. Overall, the multifaceted role of pyoverdine presents significant challenges for the clinical management of *Pseudomonas aeruginosa* infections, as it contributes to both the bacterium’s survival and virulence [31].

**Figure 7:**
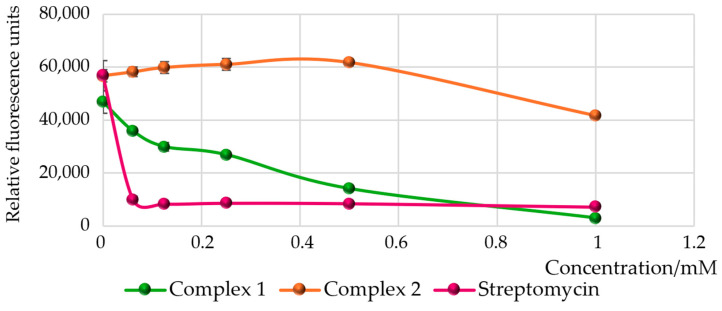
Influence of complexes **1** and **2**, and streptomycin on pyoverdine production of *P. aeruginosa* PAO1. Mean values ± standard deviations for at least three replicates are illustrated.

To assess the impact of the obtained Ru(IV) complexes on pyoverdine production by *Pseudomonas aeruginosa* PAO1, a spectrofluorometric method was employed. The results are presented in the form of a graph in Figure 7. Complex **1** was found to considerably reduce the production of pyoverdine, with decreases of 94% and 74% observed at concentrations of 1 mM and 0.5 mM, respectively, compared to the control, which was a culture medium containing bacteria. This inhibitory effect is comparable to that of the control agent, streptomycin, which decreased pyoverdine production by 86% at a concentration of 1 mM. The efficacy of complex **1** likely stems from alterations in the iron uptake system and the modulation of signaling pathways, which consequently diminish the bacteria’s survival capacity. We made similar observations for manganese compounds with heteroaromatic ligands [30]. In contrast, complex **2** at the highest concentration tested had a minimal effect on pyoverdine production relative to the control, while lower concentrations enhanced its production. This phenomenon may be caused by disturbances in the iron acquisition mechanism of bacteria under conditions of metabolic imbalance or by shifts in the fluorescence spectra of the complex formed with pyoverdine.

### 2.8. HSA Binding Studies

The elucidation of the transport mechanisms and bioavailability of the complexes within biological systems was achieved through spectroscopic methods, specifically fluorescence quenching, alongside molecular docking studies. These studies reveal the molecular interactions between human serum albumin (HSA) and complexes **1** and **2**, as well as the effects of these complexes on the structural conformation of HSA. HSA possesses the capacity to bind and transport chemically synthesized agents into systemic circulation. Therefore, understanding the interactions between HSA and the synthesized ruthenium complexes is of crucial importance. The mechanism of fluorescence quenching of HSA induced by these complexes has been elucidated through fluorescence spectral analysis. The binding constants for the ruthenium complexes with HSA were derived through mathematical analysis of the spectroscopic data. Additionally, molecular docking studies provide insights into the binding modes and molecular interactions of complex **2** with HSA.

#### 2.8.1. Human Serum Albumin Fluorescence Quenching Assay

**Figure 8:**
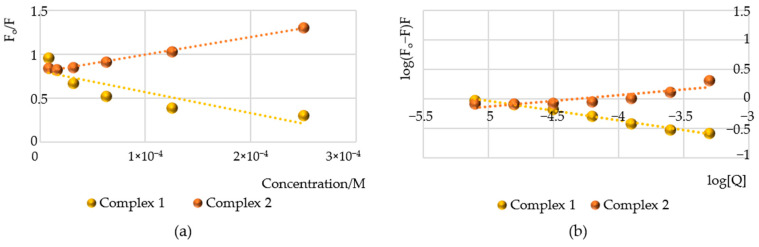
The regression plots for quenching of human serum albumin (HSA) by Ru(IV) compounds: (**a**) the Stern–Volmer plot (**b**) the double logarithm plot.

Proteins such as human serum albumin exhibit intrinsic fluorescence due to the presence of the aromatic amino acid tryptophan in their structure. This fluorescence can be quenched through the complexation of the protein with ligand molecules. Accordingly, fluorescence measurements were conducted both in the presence and absence of Ru compounds to investigate their interactions. HSA emits fluorescence at approximately 344 nm, which is primarily attributable to tryptophan residues [32]. The quenching of HSA fluorescence was continuously monitored following the addition of increasing concentrations of Ru compounds, ranging from 7.8 to 500 × 10^−6^ M. As shown in Figure S5, the fluorescence quenching intensity of HSA decreased with increasing concentrations of compounds **1** and **2** in the HSA-**1**/HSA-**2** complex, accompanied by a slight blue shift to higher energies. The spectrum for complex **1** revealed surprising results, indicating autofluorescence originating from the complex itself. These effects are clearly observed at the highest concentration tested (2.5 × 10^−4^ M), where, after the quenching of human serum albumin fluorescence, a peak is visible at approximately 375 nm. The mechanisms contributing to the quenching of human serum albumin fluorescence include both dynamic and static processes. The dynamic mechanism arises from random collisions between the excited-state complex and the protein, while the static mechanism results from the formation of a ground-state complex involving the protein. To elucidate the quenching mechanism of these complexes, the Stern–Volmer relation was employed for the construction of plots:

(1) $$\frac{F_{o}}{F}=1+K_{q}\tau_{0}\left[Q\right]=1+K_{SV}[Q]$$

where F and F_o_ represent the fluorescence intensities in the absence and in the presence of complexes **1** and **2**, K_q_ is the quenching rate constant of biomolecule, τ_0_ is the average lifetime of the biomolecule without quencher (τ_0_ = 6.2 ns), K_sv_ is Stern–Volmer constant (Figure 8a), and [Q] is the concentration of quencher. The Stern–Volmer quenching constant (K_sv_) and the rate constant for quenching (K_q_) were estimated and are presented in Table 5. Both Ru(IV) complexes exhibited a linear relationship when plotting F_o_/F against [Q], with regression coefficients (R^2^) of 0.76 or 0.97. As it is seen, the plot of F_o_/F for HSA versus [Q] ranging from 7.8 × 10^−6^ M^−1^ to 500 × 10^−6^ M^−1^ is linear for complex **1**. This may suggest that a mixed quenching mechanism, either static or dynamic is observed at these concentrations [33,34]. The binding constant of K_sv_ was calculated to be 1.98 × 10^3^ M^−1^. In the case of complex **2**, the observed increase in quenching values (F_o_/F) with rising concentration suggests the occurrence of dynamic quenching. This observation implies that the Stern–Volmer constant (K_sv_) is proportional to the concentration of the quencher, indicating a quenching mechanism associated with energy transfer to the quencher molecules. This interpretation is supported by the K_sv_ values, which increase significantly with higher concentrations of the quencher. Additionally, the quenching rate constant (K_q_ = 3.19 × 10^11^ M^−1^s^−1^) further corroborates the dynamic process. The high value of K_q_ indicates that the reactants are converted to products within a very short time frame, suggesting that the quenching process is far from equilibrium—a characteristic feature of dynamic mechanisms. According to the literature [35,36], the maximum scattering collision-quenching constant (K_q_) for various quenchers with the biopolymer is 2 × 10^10^ M^−1^s^−1^. Therefore, the quenching constants for the protein quenching process initiated by complex **2** exceed this maximum scattering collision-quenching constant. As observed in the Stern–Volmer plots (Figure 8a), the calculated value of K_sv_ is on the order of 10^3^, indicating that the interaction mechanism between HSA and complex **2** is likely initiated by dynamic collision. The binding constant (K_b_) for HSA with the Ru complexes was estimated using the double logarithmic Stern–Volmer equation:

(2) $$log\frac{F_{o}-F}{F}=logK_{b}+nlog[Q]$$

where n is Hill coefficient (describing the degree of cooperativity during the interaction of HSA with complexes) (Figure 8b). The binding constant (K_b_) and the Hill coefficient (n) were determined from the slope and intercept of the modified Stern–Volmer plot ($log\frac{F_{o}-F}{F}$ versus log[Q]) (Table 5). The Hill coefficient for both complexes was less than one, indicating a negative cooperativity between the complexes and human serum albumin. Most ligands exhibit binding constants ranging from 10^3^ to 10^6^ M^−1^, and these interactions are generally reversible. The binding constants listed in Table 5 suggest that the ruthenium complexes bind to HSA with weak affinity. The thermodynamic parameters were also calculated. Based on the binding constants, the free energy change can be estimate by the following van’t Hoff equation:

(3) $$logK_{b}=\frac{-∆H}{RT}+\frac{∆S}{R}$$

where K_b_ is the binding constant at the measuring temperatures and R is the gas constant.

The free energy change (ΔG°) can be calculated by following equation:

(4) $$∆G^{o}=∆H^{o}-T∆S^{o}=-RTlnK_{b}$$

The negative values of ΔG° indicate that the interaction processes are spontaneous (Table 5).

#### 2.8.2. Molecular Docking of the Studied Ruthenium Complexes to HSA

Initially, to find the most suitable HSA regions with the highest affinity towards the studied ruthenium complexes **1** and **2**, the whole protein surface was included in the docking simulations. In this initial test, the results were obtained only for the nitrosylruthenium complex (the reference molecule originally observed in the 7DL4 crystal structure [37]), and no results were generated for complexes **1** and **2**, suggesting very weak stabilization and lower affinity of the studied complexes to HSA compared to the reference ruthenium complex. The lack of results for complexes **1** and **2** was caused by insufficient fitting points (stabilizing interactions) between the ligand and the protein binding pocket. In the next step, to increase the likelihood of interactions, the studied ruthenium compounds (including the reference) were docked with ligands containing two identical copies of each complex. Results were obtained only for the reference and ruthenium complex **2**. For complex **1**, no stabilization of the ligand within HSA was observed, suggesting a possible dynamic exchange of this highly polar complex.

**Figure 9:**
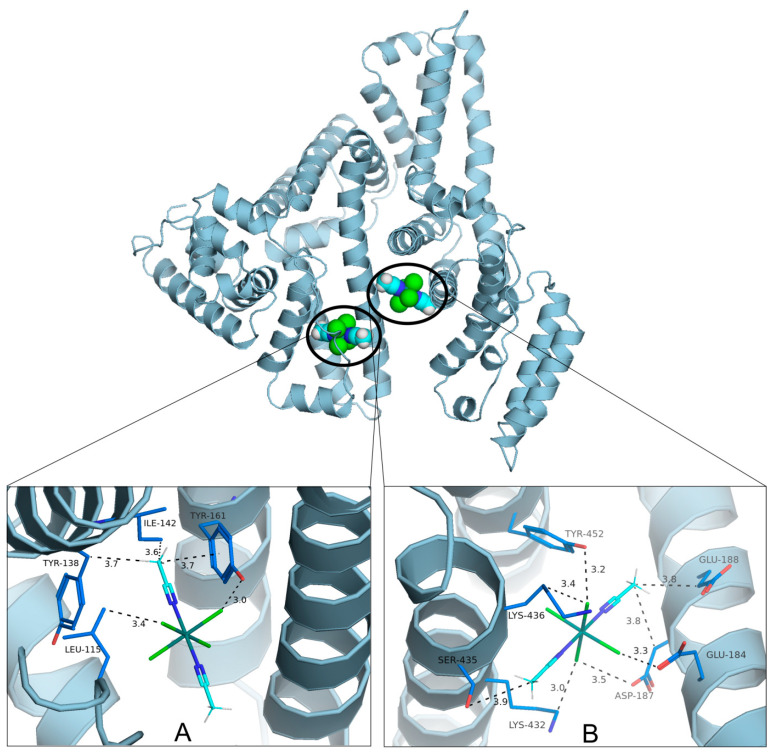
The molecular docking results for HSA and ruthenium complex **2** revealed two alternative binding regions. The ligand is stabilized by several polar and van der Waals interactions, shown in detail in parts (**A**,**B**).

In the docking studies, two possible binding regions for complex **2** were identified (Figure 9), and none of them covers albumin’s pockets defined for typical organic ligands, e.g., warfarin or ibuprofen [38]. One of the identified regions is in proximity of the area where the reference ruthenium complex binds (7DL4 crystal structure [37]). The investigated complex **2** forms an HSA/2 adduct by weak van der Waals interactions with LEU115, TYR138, ILE142, and TYR161 and one plausible O–H⋯Cl hydrogen bond with TYR161 as a donor (Figure 9A). In the second, non-specific but also more hydrophilic binding position (Figure 9B) the ligand molecule is forming a mixture of stronger and polar interactions between chloride ligands and GLU184, ASP187, and TYR452, weak hydrogen bonds with acetonitrile methyl groups as donors and GLU188, SER435 as acceptors. Moreover, LYS432 and LYS436 methylene groups form weak hydrogen bonds with chloride ligands as acceptors. Additionally, weak van der Waals contacts with ASP187 and GLU188 are observed.

The PLP scoring function value for the reference ruthenium ligand was 51.73 (two copies of the complex) and 49.94 (one copy of the complex). For complex **2**, the PLP scoring function was much lower and achieved only 27.36 (for two copies of the complex only).

As it is shown, complex **1** does not exhibit any inclination to anchor, while complex **2** shows weak docking affinity. These molecules possess greater polarity, which hinders their ability to bind stably to human serum albumin. However, they may demonstrate high or low biological activity in their free form, without requiring HSA as a potential transporter.

### 2.9. In Silico Pharmacokinetic and Drug-Likeness Predictions (ADME) for Ruthenium Complexes

Predictions indicate that the ruthenium compounds may impact cytochrome P450 isoenzymes, affecting the pharmacokinetics of other medications. Specifically, complex **2** is anticipated to inhibit CYP2C9 (Table 6), an enzyme responsible for metabolizing various substances, including steroid hormones and fatty acids, and is crucial for the breakdown of warfarin. This enzyme also plays a role in metabolizing other drugs like ibuprofen, which is used to alleviate inflammation. The anticipated physicochemical characteristics, pharmacokinetics, and drug-like properties of the studied Ru complexes are compiled in Table 7.

ADME analysis demonstrated that complex **2** is effective in crossing the blood–brain barrier (refer to Figure S6). This complex is soluble and exhibits high gastrointestinal absorption, in contrast to complex **1**, which has low solubility. The compounds under investigation are substrates for P-glycoprotein (P-gp), which limits their potential as effective treatments for multi-drug-resistant cancer cells. Unlike complex **1**, complex **2** fulfills the criteria established by Lipinski and Veber [39,40,41]. The bioavailability radar for these complexes is displayed in Figure S7. In terms of drug-like characteristics, complex **2** achieved a favorable bioavailability score of 0.55. According to the results obtained from the ProTox II service, complex **2** is classified as having class 4 toxicity, indicating it can be harmful if ingested (with an LD_50_ value between 300 and 2000). The predicted LD_50_ for complex **2** is 1000 mg/kg.

**Table 7:** Predicted physicochemical properties, pharmacokinetics, and drug-likeness features of investigated complexes **1** and **2** by the SwissADME server [42].

	Descriptor	Complex 1	Complex 2
Pharmacokinetics	GI absorption	Low	High
	BBB permeability	No	Yes
	P-gp substrate	Yes	Yes
	Water solubility (ESOL)	Poorly soluble	Soluble
Drug-Likeness	Lipiński	No, 2 violations: MW > 500, NHorOH > 5	No, 0 violation
	Veber	No, 1 violation TPSA > 140	Yes
	Bioavailability Score	0.17	0.55
	TPSA [Å^2^]	152	47.58
Physicochemical Properties (Ro5)	Number of H-Bond Acceptors	4	2
	Number of H-Bond Donors	10	0
	Log P_o/w_ (MLOGP)	−7.15	1.04
	MW [g/mol]	811.25	324.99

### 2.10. In Silico Activity Predictions

The activity of complex **2** as well as its anticancer properties in silico were predicted and presented in Table 8 and Table 9. Predicting the activity allowed us to estimate how the obtained components will theoretically affect the primary cell. The table of anticancer activity clearly shows that for complex **2** the probability of activity against Oligodendroglioma, Plasma cell myeloma, and Pancreatic carcinoma is >50%.

## 3. Materials and Methods

### 3.1. Materials

All reagents and solvents were purchased from commercial suppliers and used without further purification. The starting ruthenium(III) chloride solution (0.1 M mother solution) was prepared according to the previously described methodology [43].

### 3.2. Preparation of Complexes

#### 3.2.1. Synthesis of (Hdma)_2_(HL)_2_[Ru^IV^Cl_6_]·2Cl·2H_2_O (Complex **1**)

An ethanol solution (4 mL) of 2-hydroxymethylbenzimidazole (0.1486 g, 1 mmol) was dropped into a 0.1 M solution of ruthenium(III) chloride (5 mL, 0.5 mmol). Dimethylamine (3 mL) and hydrochloric acid (2 mL) were then added cautiously to the mixture. The solution was stirred and heated to 70–80 °C for 1.5 h. After cooling to room temperature, the resulting mixture was allowed to crystallize at −2 °C. After a few weeks, red crystals of compound **1**, suitable for X-ray structure analysis, were obtained. The product was collected by filtration and dried in air. The final yield was 36%. Found: C, 29.88; H, 4.39; N, 10.43; Calc. for RuCl_8_C_20_H_38_N_6_O_4_: C, 29.61; H, 4.72; N, 10.36%. IR: 3499 (vs), 3243 (br), 3206 (s), 3142 (vs), 3045 (m), 3032 (m), 2967 (s), 2945 (s), 2901 (ms), 2851 (ms), 2730 (ms), 1653 (w), 1624 (s), 1564 (s), 1489 (s), 1457 (vs), 1435 (vs), 1422 (vs), 1222 (vs), 1151 (s), 1082 (vs), 1022 (v), 895 (s), 839 (s), 815 (vs), 763 (vs), 668 (s). μe_ff_ = 2.49 μ_B_ for a low-spin Ru^4+^ (electron configuration of _44_Ru^4+^ [Kr] 4d^4^).

#### 3.2.2. Synthesis of [Ru^IV^Cl_4_(AN)_2_]·H_2_O (Complex **2**)

The reaction of a 0.1 M solution of ruthenium(III) chloride (5 mL, 0.5 mmol) with 11 mL of acetonitrile resulted in an orange solution. The mixture was stirred and heated to the boiling point of the solvent for one hour. After this time, the color of the starting solution changed from orange to reddish. Brown crystals for structural determination were obtained by slow evaporation of the solvent at room temperature. The crystals of complex **2** were filtered and dried in air. The compound was collected with a yield of 43%. Found: C, 14.31; H, 1.98; N, 8.26; Calc. for RuCl_4_C_4_H_8_N_2_O: C, 14.01; H, 2.35; N, 8.17%. IR: 3581 (br), 3262 (ms), 3191 (s), 3173 (s), 2981 (s), 2922 (s), 2295 (ms), 1665 (ms), 1630 (ms), 1443 (ms), 1413 (vs), 1366 (s), 1032 (s). μe_ff_ = 2.32 μ_B_ for a low-spin Ru^4+^ (electron configuration of _44_Ru^4+^ [Kr] 4d^4^).

### 3.3. Physical Measurements

Elemental analysis was performed on an Elemental Analyzer model Vario Micro Cube (Elementar, Langenselbold, Germany). The IR spectra were recorded on a Nicolet 380 FT-IR spectrophotometer (Thermo Scientific, Waltham, MA, USA) using the attenuated total reflection (ATR) technique, in the spectral range of 4000–500 cm^−1^. Electronic spectra of samples dissolved in water as well as DMSO were measured using a JASCO V-630 UV–Vis spectrophotometer (Jasco Corporation, Tokyo, Japan) with a quartz cell having a path length of 1 cm. The absorbance measurements were recorded at room temperature—ligand: 1.02 × 10^−4^ M (H_2_O), 5.20 × 10^−5^ M (DMSO); complex **1**: 9.85 × 10^−5^ M (H_2_O), 4.95 × 10^−5^ M (DMSO); complex **2**: 6.80 × 10^−5^ M (H_2_O), 4.40 × 10^−5^ M (DMSO). Magnetic measurements were carried out on a magnetic susceptibility balance (Sherwood Scientific) at room temperature by employing Gouy’s method, using Hg[Co(NCS)_4_] as a calibrant. High-resolution mass spectra (HRMS) were recorded using a Bruker micrOTOF-Q II instrument (Bruker Daltonics, Bremen, Germany) with an electrospray ionization (ESI) source. Samples were introduced into the mass spectrometer’s ion source by direct infusion via a syringe pump. The instrument operated in positive ion mode. The parameters of the ESI source were as follows: dry gas flow rate: 4.0 L/min; drying temperature: 220 °C or 180 °C; capillary voltage: 4500 V; and collision energy: 1–10 eV. Sample solutions were prepared in water (for complex **1**) and acetonitrile (for complex **2**). Electrochemical investigations (cyclic voltammetry and differential pulse voltammetry) were conducted using a Model M161E electrochemical analyzer connected to a Model M162 preamplifier and controlled with mEALab Version 2.1 software (mtm-anko, Krakow, Poland). The experiments were carried out in a three-electrode cell. A glass carbon electrode (GCE) (2 mm in diameter, A = 0.0314 cm^2^, Mineral, Warsaw, Poland) and a carbon fiber (CF) disk microelectrode (33 µm in diameter, BASi, United Kingdom) were used as working electrodes. A platinum wire and an Ag/AgCl reference electrode (containing 1 M NaCl) (both from Mineral, Warsaw, Poland) were used as auxiliary and reference electrodes, respectively. To prevent leakage of water into the solutions being tested, the reference electrode was isolated from the main electrolyte using a salt bridge with a frit of Vicor Glass. Pure argon was used to deoxygenate the solutions prior to voltammetric studies. The electrochemical investigations of the ruthenium complexes and the free ligand (concentration of compounds—1 mM) were conducted in a mixture of CH_3_CN/EtOH (3:2, *v*/*v*; Chempur, analytical grade), with 0.1 M tetrabutylammonium hexafluorophosphate (TBAPF_6_) (Fluka, electrochemical grade) as the supporting electrolyte. The measurements were performed at room temperature (25 ± 1 °C). DPV voltammograms were recorded using a pulse amplitude of 20 mV, a pulse width of 80 ms, and a scan rate of 20 mV s^−1^.

### 3.4. Crystal Structure Determination

Single crystal X-ray diffraction measurements of complexes **1** and **2** were collected at 293 K on a KappaCCD diffractometer (Nonius) using monochromatic graphite MoKα radiation (λ = 0.71073 Å). Cell refinement and data reduction were performed using firmware [44,45]. Corrections for Lorentz, polarization, and absorption effects [44,45] were applied. The structures were solved by direct methods using the program package SIR-92 [46] and refined using a full-matrix least square procedure on F^2^ using SHELXL-2019/2 [47,48]. All hydrogen atoms attached to carbon atoms were placed in idealized geometries and refined using a driving model with U_iso_ (H) fixed at 1.2 U_eq_ (C). The crystal structures of the studied compounds show the presence of water molecules in the lattice. Unfortunately, based on the differential Fourier map, it was not possible to determine the position of hydrogen atoms next to oxygen atoms in water molecules, which generates the appearance of B alerts: “PLAT306_ALERT_2_B Isolated Oxygen Atom (H-atoms Missing)” in the checkcif files for both crystal structures. The diffraction experiment for the structure of compound **2** was performed on a slightly racemically twinned crystal, exhibiting a component ratio of 0.95:0.05. The crystallographic data and detailed information on the structure solution and refinement for the ruthenium complexes **1** and **2** are given in Table S4 The figures were made using DIAMOND version 3.1 f [49] software. CCDC 2388592 and 2388593 contain the supplementary crystallographic data for **1** and **2**, respectively. These data can be obtained free of charge from The Cambridge Crystallographic Data Centre via www.ccdc.cam.ac.uk/data_request/cif (accessed on 6 November 2024).

### 3.5. Hirshfeld Surface Analysis

Calculations of molecular Hirshfeld surfaces were conducted using the Crystal Explorer software package, version 21.5 [50]. Upon inputting the .cif file corresponding to the title compounds into the Crystal Explorer program, version 21.5, all bond lengths to hydrogen atoms were automatically adjusted to conform to standard neutron scattering values (CH = 1.083 Å). The analysis of the Hirshfeld surfaces incorporated the descriptor *d*_norm_ which represents the normalized distance to the nearest atomic nuclei, as well as the *shape index* [51,52]. The computational procedures and analytical details are elaborated in reference [53]. The molecular Hirshfeld surfaces for complexes **1** and **2** were produced at the standard (high) surface resolution, with the 3D *d*_norm_ surfaces mapped over a fixed color scale, where red indicates values ranging from −0.6331 Å (for complex **1**) and −1.1896 Å (for complex **2**) to blue, representing values of 1.1951 Å (for complex **1**) and 1.2599 Å (for complex **2**). The *shape index* was represented within a color range from −1 to 1 for both complexes. This color coding effectively illustrates the relative “strength” of various types of intermolecular interactions present. Additionally, fingerprint plots provide a quantitative assessment of the intermolecular interactions occurring on the surface.

### 3.6. Microbiological Studies

#### 3.6.1. Bacterial Strains and Cultivation

The PAO1 strain of *Pseudomonas aeruginosa* was obtained from the International *Pseudomonas aeruginosa* Reference Panel, housed at the Belgian Co-ordinated Collection of Microorganisms (BCCM)/LMG Bacteria Collection at Ghent University, Belgium (http://bccm.belspo.be/about-us/bccm-lmg; accessed on 12 December 2024) [54]. The strains used in the experiment, *Staphylococcus aureus* ATCC 6538P and *Escherichia coli* ATCC 8739, are representative examples of Gram-positive and Gram-negative bacteria, respectively. The bacteria were grown overnight at 37 °C with shaking at 160 r.p.m. using an Ecotron incubator (Infors HT, Basel, Switzerland), in Trypticase Soy Broth (TSB) medium (Biocorp, Warsaw, Poland). Following this, the cultures were diluted in fresh medium at a 1:100 ratio, resulting in bacterial cell densities of approximately 10^7^ CFU/mL, and supplemented with the compounds being tested. The bacteria were then subjected to various assays.

#### 3.6.2. Minimum Inhibitory Concentration

The Minimal Inhibitory Concentration (MIC) was assessed using a dilution technique with 96-well flat-bottom transparent microtiter plates (Greiner, Monroe, NC, USA). An overnight bacterial culture (*S. aureus*, *E. coli*, *P. aeruginosa* PAO1) was diluted 1:100 (0.5 McFarland turbidity) in fresh TSB medium and treated with aqueous solutions of Ru(IV) complexes at concentrations ranging from 1 mM to 0.0625 mM, along with resazurin (0.02 mg/mL) as a growth indicator [55]. The plates were then incubated overnight at 37 °C with shaking at 160 r.p.m. For each test batch, two control tubes were included: a negative control (bacterial culture in the medium without any growth inhibitor) and a positive control (antibiotic control—streptomycin). The MIC was defined as the lowest concentration of the tested compounds that prevented any microbial growth.

#### 3.6.3. Inhibition of *P. aeruginosa* PAO1 Biofilm Formation

An overnight culture of *Pseudomonas aeruginosa* PAO1, along with the examined complexes, was subjected to assays for adherence and biofilm formation. The level of adhesion was assessed by measuring the number of adherent cells on 96-well transparent flat-bottom microtiter plates (Greiner, Monroe, NC, USA) using crystal violet staining at a concentration of 0.01% (*w*/*v*). The overnight culture was diluted 1:100 in fresh TSB medium (180 μL) and transferred into the 96-well microtiter plates. Serial dilutions of the compounds under investigation were prepared, covering a concentration range from 0.0625 mM to 1 mM. For comparison, serial dilutions of streptomycin at the same concentrations were included as a control. The plates were incubated overnight at 37 °C. After incubation, any remaining planktonic cells were removed by washing the plate with distilled water. The plates were then allowed to dry, and 200 μL of crystal violet solution was added, followed by a 15 min incubation at room temperature. The crystal violet solution was subsequently removed, and the plates were washed again with distilled water, repeating this step three times. To extract the crystal violet from the adhered cells, the wells were filled with 96% ethanol and incubated for 15 min at RT, after which absorbance was measured at λ = 595 nm using an Infinite M200PRO microplate reader (Tecan, Männedorf, Switzerland). The absorbance data were expressed in arbitrary units and converted to percentages to facilitate comparisons of results across different experiments.

#### 3.6.4. Pyoverdine Determination Assay

The tests assessed the effect of complexes 1 and 2 on the reduction of pyoverdine, at the concentrations of tested compounds ranging from 1 mM to 0.0625 mM. Detection of fluorescent pyoverdine was performed photometrically at an excitation wavelength of λ = 398 nm and an emission wavelength of λ = 455 nm, in accordance with previous studies [56]. Measurements were conducted using the Infinite M200PRO device (Tecan, Männedorf, Switzerland). Streptomycin was used as a negative control. The experiments were carried out in triplicate using 96-well black microtiter plates (Greiner, Monroe, NC, USA) for a duration of 24 h in a cell-free culture medium (TSB).

### 3.7. Human Serum Albumin (HSA) Fluorescence Quenching Assay

The fluorescence quenching assay was conducted in accordance with a previously established protocol [56] to investigate the interactions between ruthenium(IV) complexes and human serum albumin (HSA). The concentration of HSA was maintained at 10 µM, and its fluorescence quenching was assessed. Fluorescence spectra were recorded at a temperature of 310 K over the range of 320 to 400 nm upon excitation at 280 nm for human serum albumin. Solutions containing HSA were treated with various concentrations of ruthenium compounds (0, 7.8, 15.6, 31.3, 62.5, 125, 250, or 500 µM), after which the fluorescence intensity was recorded at a constant emission wavelength of λ = 344 nm, with an excitation wavelength of λ = 280 nm. All measurements were performed using the Infinite M200PRO microplate reader (Tecan, Männedorf, Switzerland).

### 3.8. Molecular Docking Procedures

The crystal structure of HSA (Human Serum Albumin) with nitrosylruthenium complex adduct (PDB ID: 7DL4 [37]) was downloaded from the Protein Data Bank (PDB) [57]. The initial geometries of the studied Ru^4+^ complexes were taken from the final refinement generated .cif files. Ligand structures were generated in two ways: structures containing single or two ruthenium complexes, for which the docking procedure was performed. The protein and ligand structure preparation was conducted using Maestro 13.9 [58] and Mercury 2023.3.0 programs [59]. Molecular docking experiments were performed in a semi-flexible mode using GOLD 2023.3.0 (Genetic Optimisation for Ligand Docking) [60].

Molecular docking studies were conducted in two steps. First, the docking region was expanded to a radius of 50 Å to include all atoms of the HSA. This procedure allowed a search for the most prominent binding sites for the investigated ruthenium complexes. The native nitrosylruthenium complex of the 7DL4 structure was re-docked as a reference ligand. The number of genetic algorithm runs was set to 50 for each docked compound.

In the second stage, the main docking region identified during the previous experiment was selected. The docking sphere was centered at Val455, including all atoms within the 25 Å radius. The number of genetic algorithm runs was set to 20 for each compound. Every step was evaluated based on the reference nitrosylruthenium complex results.

The ChemPLP empirical scoring function [61] was applied in all docking procedures,. Obtained molecular docking results were analyzed and visualized with PyMOL [62] and Protein-Ligand Interaction Profiler (PLIP) server [63].

### 3.9. ADME Analysis

The ADME (Absorption, Distribution, Metabolism, and Excretion) analysis was conducted using the SwissADME service [42] (Swiss Institute of Bioinformatics, 2024, https://www.sib.swiss/, accessed 12 December 2024). This free online tool calculates physicochemical descriptors and predicts ADME parameters, pharmacokinetic properties, drug-like characteristics, and medicinal chemistry compatibility for one or more small molecules to aid in drug discovery. Additionally, the ProTOX II service [64,65,66] was utilized to assess the toxicity of the ruthenium compounds.

### 3.10. Activity Predictions

The activity of the complexes was evaluated using the websites http://www.way2drug.com/PASSonline/ and http://www.way2drug.com/Cell-line/ (accessed on 16 December 2024). PASS Online is capable of predicting types of biological activities, which include pharmacological effects, mechanisms of action, toxic and adverse effects, interactions with metabolic enzymes and transporters, and effects on gene expression, among others. It offers a reasonable level of accuracy in its predictions [67]. CLC-Pred (Cell Line Cytotoxicity Predictor) is an online tool designed for the in silico prediction of the cytotoxic effects of chemical compounds on both non-transformed and cancer cell lines based on their structural formulas [68].

## 4. Conclusions

In this study, two new chloride ruthenium complexes in high oxidation states were synthesized and characterized by an analytical method. Their crystal structures were also determined by SC-X-ray diffraction analysis. In the obtained complexes, the metal in +IV oxidation state occurs in the complex in the octahedral geometry. Similar to observations in our previous studies, chloride complex **1** was formed instead of a chelating complex. The hydrogen bonds in the crystal structures of the complexes (notably O–H⋯O, N–H⋯Cl, and C–H⋯Cl) along with π⋯π stacking interactions, play a significant role in stabilizing the crystal architecture. In crystal packing, dma plays a similar role to ethanol and is engaged in molecular clips. Importantly, Hirshfeld surface analysis confirmed the predominance of Cl⋯H, Cl⋯O and H⋯H contacts. The results of cyclic voltammetry and magnetic measurements confirmed the high +IV oxidation state of Ru in both complexes. Ruthenium complex **1** exhibits promising properties that could be harnessed in the fight against bacterial infections by *P. aeruginosa*, as it demonstrates strong oxidizing properties. These valuable properties could be crucial for explaining the mechanism of action and identifying biological targets. The relationship between *E*_1/2_ and the level of biofilm inhibition shows a decreasing trend: as the half-wave potential increases, the biological activity decreases. Based on the obtained results, it can be concluded that high-valent Ru complexes may induce a cellular response similar to oxidative stress in bacterial cells.

Biological studies of the tested Ru(IV) complexes have confirmed the significantly higher anti-biofilm activity of complex **1** against *Pseudomonas aeruginosa* PAO1 compared to complex **2**. Moreover, the activity against biofilm formation was found to be greater than that against planktonic bacteria, indicating the selectivity of the complexes. Importantly, complex **1** significantly contributes to the reduction of pyoverdine secretion, which, in turn, leads to a decrease in the virulence of the *P. aeruginosa* PAO1 strain. In contrast, the inhibition of pyoverdine secretion by complex **2**, even at the highest concentration, is low. Additionally, molecular interactions between human serum albumin and the investigated Ru complexes were studied by spectroscopic and molecular docking approaches. The applied spectroscopic method enabled us to specifically assess the interactions occurring around the protein fluorophores, especially tryptophan in HSA. The results showed that the complexes quench the fluorescence emitted by HSA through a dynamic/mixed mechanism (by collisions between the excited-state complex and the protein or by the formation of the drug–protein complex), as illustrated in the Stern–Volmer plot. The binding parameters on the order of 10^1^, determined by SV and DLSV equations, demonstrate weak affinity to HSA. These findings correlate with molecular docking studies. Two potential binding sites for complex **2** were identified, neither of which overlaps with the binding pockets of albumin that are typically associated with organic ligands. The molecular docking study identified weak hydrogen bonds, polar interactions, and Van der Waals interactions as the main forces involved in the binding of HSA to complex **2**.

The conducted research on the ruthenium(IV) complexes has demonstrated that these compounds may have potential applications as anti-biofilm agents. This is particularly significant in light of the growing problem of antibiotic resistance. The evaluation of the anti-biofilm activity of these complexes and their impact on virulence factors, such as pyoverdine, suggests their potential in combating bacterial biofilm formation. Therefore, further investigations into their mechanism of action and safety profile are essential for fully understanding and establishing their therapeutic efficacy.

## Figures and Tables

**Table 1:** UV–Vis spectroscopic data for the ligand and the ruthenium complexes.

Compound	Solvent	Transition λ, nm (ε, dm^3^/mol∙cm)		
		π → π*	LMCT	*d* *–d*
Ligand	H_2_O	214 (9436), 244 (8697), 273 (8506), 279 (7917)	–	–
	DMSO	276 (8032), 283 (7478)	–	–
Complex **1**	H_2_O	215 (10,219), 233 (6462), 268 (8116), 274 (7634)	325 (678), 360 (476)	491 (115)
	DMSO	271 (14,074), 277 (14,637)	418 (4429)	498 (497)
Complex **2**	H_2_O	218 (14,261), 241 (8751), 295 (1693)	379 (4113), 392 (4562)	454 (310)
	DMSO	264 (11,987), 302 (2976)	400 (9473), 415 (10,457)	477 (982)

**Table 2:** Selected bond lengths (Å) and angles (°) for complex **1**.

Bond Lengths (Å)			
Ru1–Cl2	2.3687(6)	Ru1–Cl1	2.3846(6)
Ru1–Cl3	2.3721(6)		
Valence angles (°)			
Cl2–Ru1–Cl2_(−x,−y−1,−z+1)_	180.0	Cl2–Ru1–Cl1	89.79(2)
Cl2–Ru1–Cl3	90.25(2)	Cl3–Ru1–Cl1	90.03(2)
Cl3–Ru1–Cl3_(−x,−y−1,−z+1)_	180.00(3)	Cl1–Ru1–Cl1_(−x,−y−1,−z+1)_	180.0

**Table 3:** Selected bond lengths (Å) and angles (°) for complex **2**.

Bond Lengths (Å)			
Ru1–N13	2.024(8)	Ru2–N23	2.031(8)
Ru1–N3	2.039(8)	Ru2–Cl6	2.335(3)
Ru1–Cl4	2.330(2)	Ru2–Cl5	2.347(3)
Ru1–Cl1	2.349(3)	Ru3–N33	2.028(9)
Ru1–Cl2	2.369(2)	Ru3–Cl8	2.342(3)
Ru1–Cl3	2.370(3)	Ru3–Cl7	2.345(3)
Valence angles (°)			
N13–Ru1–N3	178.8(3)	Cl4–Ru1–Cl2	177.74(9)
N13–Ru1–Cl4	89.5(2)	Cl1–Ru1–Cl2	90.16(9)
N3–Ru1–Cl4	90.4(2)	N13–Ru1–Cl3	91.9(2)
N13–Ru1–Cl1	90.0(2)	N3–Ru1–Cl3	89.3(3)
N3–Ru1–Cl1	88.8(3)	Cl4–Ru1–Cl3	89.75(9)
Cl4–Ru1–Cl1	91.06(9)	Cl1–Ru1–Cl3	177.91(9)
N13–Ru1–Cl2	88.6(2)	Cl2–Ru1–Cl3	89.09(9)
N3–Ru1–Cl2	91.5(2)		
N23_(−x+1,−y,−z)_–Ru2–N23	180.0	Cl6–Ru2–Cl5	90.60(1)
N23–Ru2–Cl6_(−x+1,−y,−z)_	92.0(3)	N23–Ru2–Cl5_(−x+1,−y,−z)_	87.1(3)
N23–Ru2–Cl6	88.0(3)	Cl6–Ru2–Cl5_(−x+1,−y,−z)_	89.40(1)
Cl6_(−x+1,−y,−z)_–Ru2–Cl6	180.00(8)	Cl5–Ru2–Cl5_(−x+1,−y,−z)_	180.0
N23–Ru2–Cl5	92.9(3)		
N33–Ru3–N33_(−x+2,−y+1,−z)_	180.0	Cl8–Ru3–Cl7_(−x+2,−y+1,−z)_	89.18(1)
N33–Ru3–Cl8	89.4(3)	N33–Ru3–Cl7	91.8(3)
N33–Ru3–Cl8_(−x+2,−y+1,−z)_	90.6(3)	Cl8–Ru3–Cl7	90.83(1)
Cl8–Ru3–Cl8_(−x+2,−y+1,−z)_	180.0	Cl7_(−x+2,−y+1,−z)_–Ru3–Cl7	180.0
N33–Ru3–Cl7_(−x+2,−y+1,−z)_	88.2(3)		

**Table 4:** Cyclic voltammetry data [potentials vs. Ag/AgCl in V] of ruthenium(IV) complexes.

Complex	Scan Rate mV/s	Ru(IV) ↔ Ru(III)				Ru(III) ↔ Ru(II)				Ru(II) ↔ Ru(I)			
		*E*_pa_	*E*_pc_	Δ*E*_p_	*E*_1/2_	*E*_pa_	*E*_pc_	Δ*E*_p_	*E*_1/2_	*E*_pa_	*E*_pc_	Δ*E*_p_	*E*_1/2_
**1**	25	0.198	0.039	0.159	0.119	~−0.065	~−0.215	#	#	~−0.065	~−0.215	#	#
	50	0.200	0.037	0.163	0.119	~−0.055	~−0.210	#	#	~−0.055	~−0.210	#	#
	100	0.201	0.035	0.166	0.118	~−0.045	~−0.200	#	#	~−0.045	~−0.200	#	#
**2**	25	0.195	0.140	0.055	0.168	~−0.070	~−0.130	~0.060	~−0.100	−0.250	−0.370	0.120	−0.310
	50	0.196	0.139	0.057	0.168	~−0.085	~−0.150	~0.065	~−0.118	−0.260	−0.380	0.120	−0.320
	100	0.198	0.138	0.060	0.168	~−0.090	~−0.160	~0.070	~−0.125	−0.270	−0.395	0.125	−0.333

Δ*E*_p_ = *E*_pa_ − *E*_pc_, *E*_1/2_ = ½(*E*_pc_ + *E*_pa_). #—not determinable.

**Table 5:** The Stern–Volmer constants K_sv_ and the quenching rate constants K_q_, binding constants K_b_, number of binding sites n, and thermodynamic parameter for the interaction of HSA with studied Ru compounds.

Compound	Quenching		Binding		Thermodynamic
	K_SV_ [M^−1^]	K_q_ [M^−1^ s^−1^]	K_b_ [M^−1^]	n	$∆G^{o}$ [kJ mol^−1^]
**1**	1.98 × 10^3^	3.19 × 10^11^	4.81 × 10^1^	0.33	−9.59
**2**	2.37 × 10^3^	3.82 × 10^11^	7.05	0.23	−4.84

**Table 6:** Activity prediction results for the complex **2**.

Classification	Target/Shorthand	Prediction	Probability
Metabolism	Cytochrome CYP1A2	Inactive	0.66
	Cytochrome CYP2C19	Inactive	0.68
	Cytochrome CYP2C9	Active	0.50
	Cytochrome CYP2D6	Inactive	0.53
	Cytochrome CYP3A4	Inactive	0.70
	Cytochrome CYP2E1	Inactive	0.97

**Table 8:** Cancer cell line prediction results for the complex **2**.

P_a_	P_i_	Cell-Line	Cell-Line Name	Tissue
0752	0.006	HS683	Oligodendroglioma	Brain
0.523	0.006	U-266	Plasma cell myeloma	Blood
0.513	0.005	YAPC	Pancreatic carcinoma	Pancreas

P_a_ denotes probability “to be active” and P_i_ denotes probability “to be inactive”.

**Table 9:** Non-tumor cell line prediction results for the complex **2**.

P_a_	P_i_	Cell-Line	Cell-Line Name	Tissue
0.259	0.003	IMR-90	Embryonic lung fibroblast	Brain
0.250	0.085	MRC5	Plasma cell myeloma	Blood
0.213	0.094	HEK293	Pancreatic carcinoma	Pancreas

P_a_ denotes probability “to be active” and P_i_ denotes probability “to be inactive”.

## Data Availability

Data are contained within the article and Supplementary Materials.

## Supplementary Materials

The following supporting information can be downloaded at https://www.mdpi.com/article/10.3390/molecules30030564/s1, Figure S1: UV–Vis spectra of 2-hydroxymethylbenzimidazole and complexes **1** and **2**, recorded in distilled water (a,c) and in dimethyl sulfoxide (b,d); Figure S2: Comparison of UV–Vis spectra of complex **1** in H_2_O and DMSO (a) and complex **2** in H_2_O and DMSO (b); Figure S3: UV–Vis spectra of complexes **1** and **2** in aqueous solution (a,c) and in dimethyl sulfoxide solution (b,d), measured immediately after preparation and after 24 h. Figure S4: DPV curves for complex **1** recorded over time in the potential range from −0.1 to 0.4 V; Figure S5: The fluorescence spectra of HSA in the absence or presence of complexes **1** (a) and **2** (b) at a temperature of 310 K. The concentration of HSA and the complexes were: 10 μM and 15.6–250 μM, respectively. Figure S6: Bioavailability radar for complex **1** (a) and **2** (b); Figure S7: Boiled-Egg diagram for Ru compounds **1** and **2** illustrating lipophilicity (WLOGP) and polarity (TPSA). The white region represents human intestinal absorption, while the yellow area indicates permeation through the blood-brain barrier (generated by the SwissADME server accessed on 12 December 2024); Table S1: The characteristic IR absorption frequencies (cm^−1^) of the ligand and the ruthenium complexes; Table S2: Geometry of classical hydrogen bonds and supramolecular interactions of C–H⋯Cl type for complex **1** and **2**. (Å, °); Table S3: Results of the minimum inhibitory concentration (MIC) for the compounds evaluated, expressed in mM and μg/mL; Table S4: Crystallographic data and structure refinement details for the Ru complexes.
