# Supplementary material for: Chloride and Acetonitrile Ruthenium(IV) Complexes: Crystal Architecture, Chemical Characterization, Antibiofilm Activity, and Bioavailability in Biological Systems
Source: Molecules. 2025 Jan 26;30(3):564. doi: 10.3390/molecules30030564 (PMC11820517; doi:10.3390/molecules30030564)
Supplement: Supplementary file 1 [file molecules-30-00564-s001.zip › molecules-3412762-supplementary.pdf]

Electronic Supplementary Information

Chloride and Acetonitrile Ruthenium(IV) Complexes: Crystal Architecture, Chemical Characterization, Antibiofilm Activity, and Bioavailability in Biological Systems

Agnieszka Jabłońska-Wawrzycka[1,*], Patrycja Rogala[1,*], Grzegorz Czerwonka[2], Maciej Hodorowicz[3], Justyna Kalinowska-Tłuścik[3] and Marta Karpiel[3,4]

[1] *Institute of Chemistry, Jan Kochanowski University, 7 Uniwersytecka Str., 25-406 Kielce, Poland;*
[2] *Institute of Biology, Jan Kochanowski University, 7 Uniwersytecka Str., 25-406 Kielce, Poland;*
[3] *Faculty of Chemistry, Jagiellonian University, 2 Gronostajowa Str., 30-387 Cracow, Poland;*
[4] *Doctoral School of Exact and Natural Sciences, Jagiellonian University, 11 Lojasiewicza Str., 30-348 Cracow, Poland.*

Correspondence: agnieszka.jablonska-wawrzycka@ujk.edu.pl; patrycja.rogala@ujk.edu.pl

CONTENTS

1. Supporting tables……………………………………………........S2 – S5

2. Supporting figures………………………………………….......S6 – S10

1. Supporting tables

Table S1. The characteristic IR absorption frequencies (cm^{-1}) of the ligand and the ruthenium complexes.

Assignment	Ligand	Complex 1	Complex 2
$\nu_{O\text{-}H}$ (H_2O, L)	– ; 3230	3499; 3243	3581; –
$\nu_{N\text{-}H}$	3099	3206; 3142	–
$\nu_{C\text{-}H\ aromatic}$	3059, 3033	3045, 3032	–
$\nu_{C\text{-}H\ aliph.}$	2745	2967, 2945, 2901, 2851, 2730	2981, 2922
$\nu_{C\equiv N}$	–	–	2295
$\nu_{C=C,\ C=N\ skeletal\ ring}$	1621, 1589, 1487, 1456, 1437	1653, 1624, 1564, 1489, 1457, 1435, 1422	–

Table S2. Geometry of classical hydrogen bonds and supramolecular interactions of C–H···Cl type for complex **1** and **2**. (Å, °).

Complex	D–H···A	D–H (Å)	H···A / (Å)	D···A/ (Å)	∠D-H···A/(°)
**1**	O11–H11···O1	0.82	1.95	2.768(6)	172.9
	C12–H12B···Cl1$_{(x+1,y,z–1)}$	0.96	2.95	3.595(3)	125.7
	N1–H1···Cl2$_{(–x,–y,–z+1)}$	0.78(4)	2.78(4)	3.342(3)	131(4)
	N1–H1···Cl3$_{(x,y+1,z)}$	0.78(4)	2.52(4)	3.184(3)	145(4)
	N11–H11B···Cl1$_{(–x+1,–y,–z)}$	0.87(5)	2.75(5)	3.432(3)	136(4)
	N11–H11B···Cl2$_{(–x+1,–y,–z)}$	0.87(5)	2.71(5)	3.370(3)	134(4)
	N11–H11B···Cl3$_{(–x+1,–y,–z)}$	0.87(5)	2.62(5)	3.254(3)	131(4)
	N11–H11A···Cl2$_{(x,y+1,z–1)}$	0.97(6)	2.49(6)	3.264(3)	136(5)
	N11–H11A···Cl3$_{(x,y+1,z–1)}$	0.97(6)	2.61(6)	3.405(3)	139(5)
	N3–H3···Cl4	0.90(5)	2.13(5)	2.977(3)	156(5)
**2**	C1–H1C···Cl3$_{(–x,–y+1,–z+1)}$	0.96	2.97	3.580(1)	122.6
	C1–H1B···Cl7$_{(x,y,z+1)}$	0.96	2.94	3.606(1)	127.3
	C11–H11A···Cl5$_{(–x,–y,–z)}$	0.96	2.77	3.709(12)	166.4
	C11–H11B···Cl1$_{(–x+1,–y,–z+1)}$	0.96	2.95	3.558(12)	122.8
	C11–H11B···Cl4$_{(–x,–y,–z+1)}$	0.96	2.88	3.456(11)	119.2
	C11–H11B···Cl8$_{(–x+1,–y,–z)}$	0.96	2.87	3.560(10)	129.3
	C11–H11C···Cl6	0.96	2.84	3.678(10)	147.0
	C31–H31B···Cl3$_{(x+1,y,z)}$	0.96	2.82	3.490(11)	127.7
	C31–H31A···Cl2$_{(x+1,y,z)}$	0.96	2.93	3.737(12)	142.4
	C31–H31C···Cl5$_{(x+1,y,z)}$	0.96	2.74	3.235(12)	112.5
	C21–H21B···Cl1	0.96	2.92	3.732(11)	143.1
	C21–H21A···Cl3$_{(x+1,y,z)}$	0.96	2.94	3.742 (11)	141.2
	C21–H21A···Cl4$_{(x+1,y,z)}$	0.96	2.95	3.741(10)	140.8
	C21–H21C···Cl1$_{(–x+1,–y,–z+1)}$	0.96	2.86	3.726(11)	150.2

Table S3. Results of the minimum inhibitory concentration (MIC) for the compounds evaluated, expressed in mM and μg/mL.

**Compound**	**BACTERIA**					
	S. aureus		***E. coli***		***P. aeruginosa***	
	mM	μg/mL	mM	μg/mL	mM	μg/mL
$RuCl_3 \cdot xH_2O$	> 1	> 207	> 1	> 207	> 1	> 207
L	> 1	> 148	> 1	> 148	> 1	> 148
HL	> 1	> 149	> 1	> 149	> 1	> 149
Complex **1**	> 1	> 811	1	811	1	811
Complex **2**	> 1	> 343	> 1	> 343	> 1	> 343
Streptomycin	0.0625	36	0.125	73	0.0625	36

Table S4. Crystallographic data and structure refinement details for the Ru complexes.

	**1**	**2**
Empirical formula	$C_{20}H_{34}Cl_8N_6O_4Ru$	$C_4H_6Cl_4N_2ORu$
Formula weight (g/mol)	807.20	340.98
Temperature (K)	293(2)	
Wavelength of MoKα radiation (Å)	0.71073	
Crystal system, space group	triclinic, $P\bar{1}$	triclinic, $P\bar{1}$
Unit cel dimensions		
a (Å)	7.02670(1)	8.0315(5)
b (Å)	9.4347(2)	11.9357(8)
c (Å)	13.3900(3)	12.9305(7)
α (°)	81.904(2)	102.647(4)
β (°)	77.191(2)	105.403(4)
γ (°)	71.219(2)	92.308(4)
Volume (Å^3)	817.19(3)	1159.62(1)
Z, density (calculated) (Mg/m^3)	1; 1.640	4; 1.953
Absorption coefficient (mm^{-1})	1.170	2.233
F (000)	408	656
Crystal size (mm)	0.20 × 0.10 × 0.01	0.30 × 0.30 × 0.20
Theta range for data collection (°)	2.672 – 30.545	2.645 – 27.430
Index ranges	–10 ≤ h ≤ 9,<br>–13 ≤ k ≤ 13,<br>–19 ≤ l ≤ 18	–10 ≤ h ≤ 10,<br>–15 ≤ k ≤ 14,<br>–16 ≤ l ≤ 16
Reflections collected/independent<br>[I> 2sigma(I)]	43901/4744<br>[R_{int} = 0.0569]	8685/5201<br>[R_{int} = 0.0327]
Completeness to θ (%)	θ = 25.242°; 99.8	θ = 25.242°; 98.9
Refinement method	Full-matrix least-squares on F^2	
Data/restraints/parameters	4744/0/197	5201/0/225
Goodness-of-fit on F^2	1.066	1.172
Final R indices [I>2sigma(I)]	R_1 = 0.0418,<br>wR_2 = 0.1071	R_1 = 0.0757,<br>wR_2 = 0.1968
R indices (all data)	R_1 = 0.0485,<br>wR_2 = 0.1135	R_1 = 0.0908,<br>wR_2 = 0.2065
Largest diff. peak and hole (e·Å^{-3})	1.036 and –2.233	4.038 and –1.759

2. Supporting figures

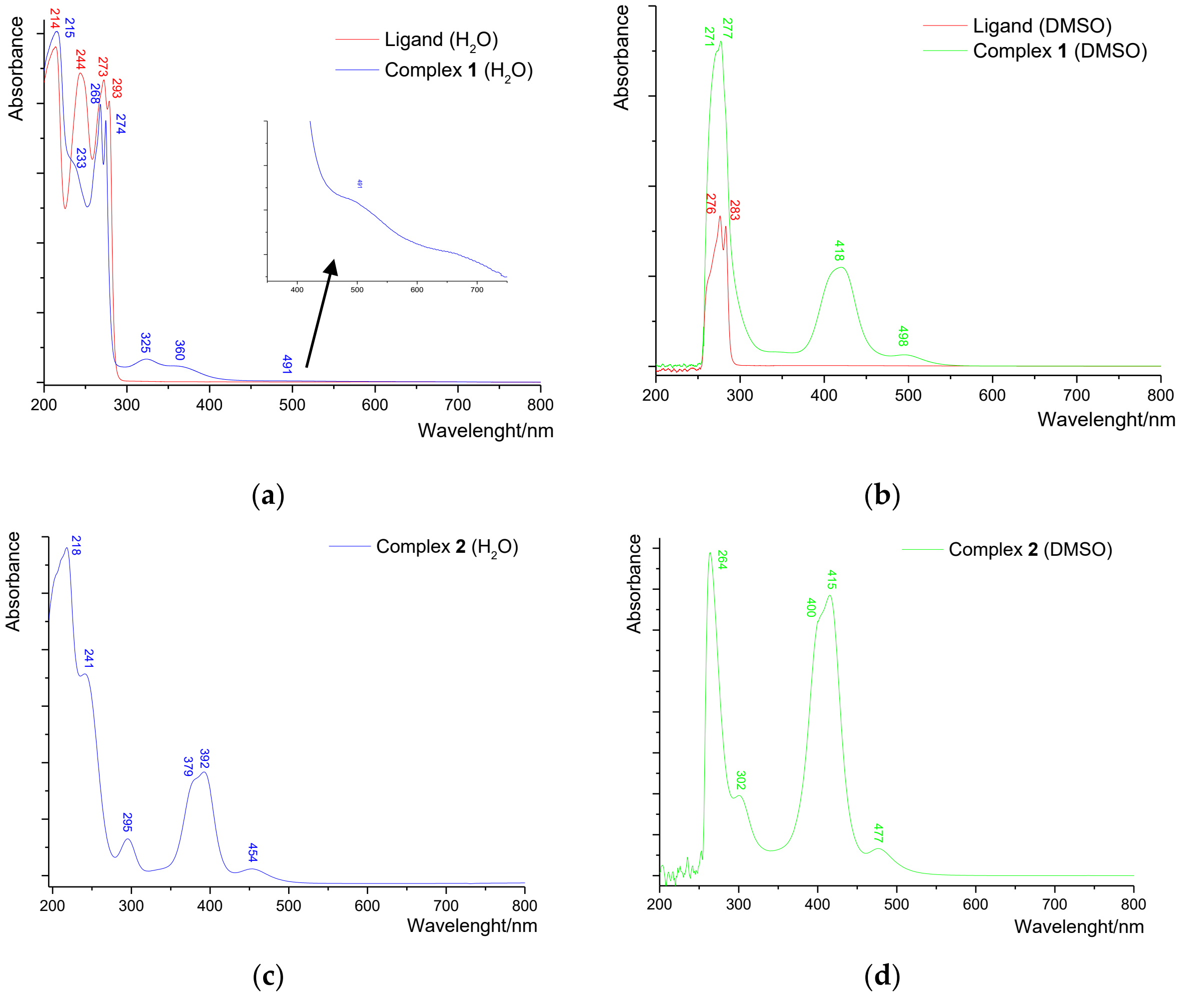

Figure S1. UV–Vis spectra of 2-hydroxymethylbenzimidazole and complexes **1** and **2**, recorded in distilled water (**a**, **c**) and in dimethyl sulfoxide (**b**, **d**).

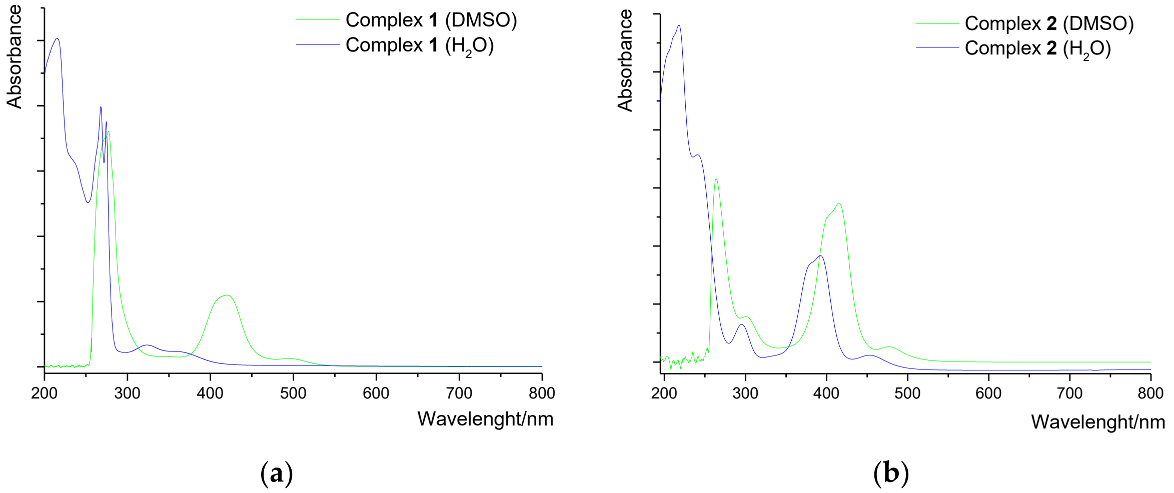

Figure S2. Comparison of UV-Vis spectra of complex **1** in H_2O and DMSO (**a**) and complex **2** in H_2O and DMSO (**b**).

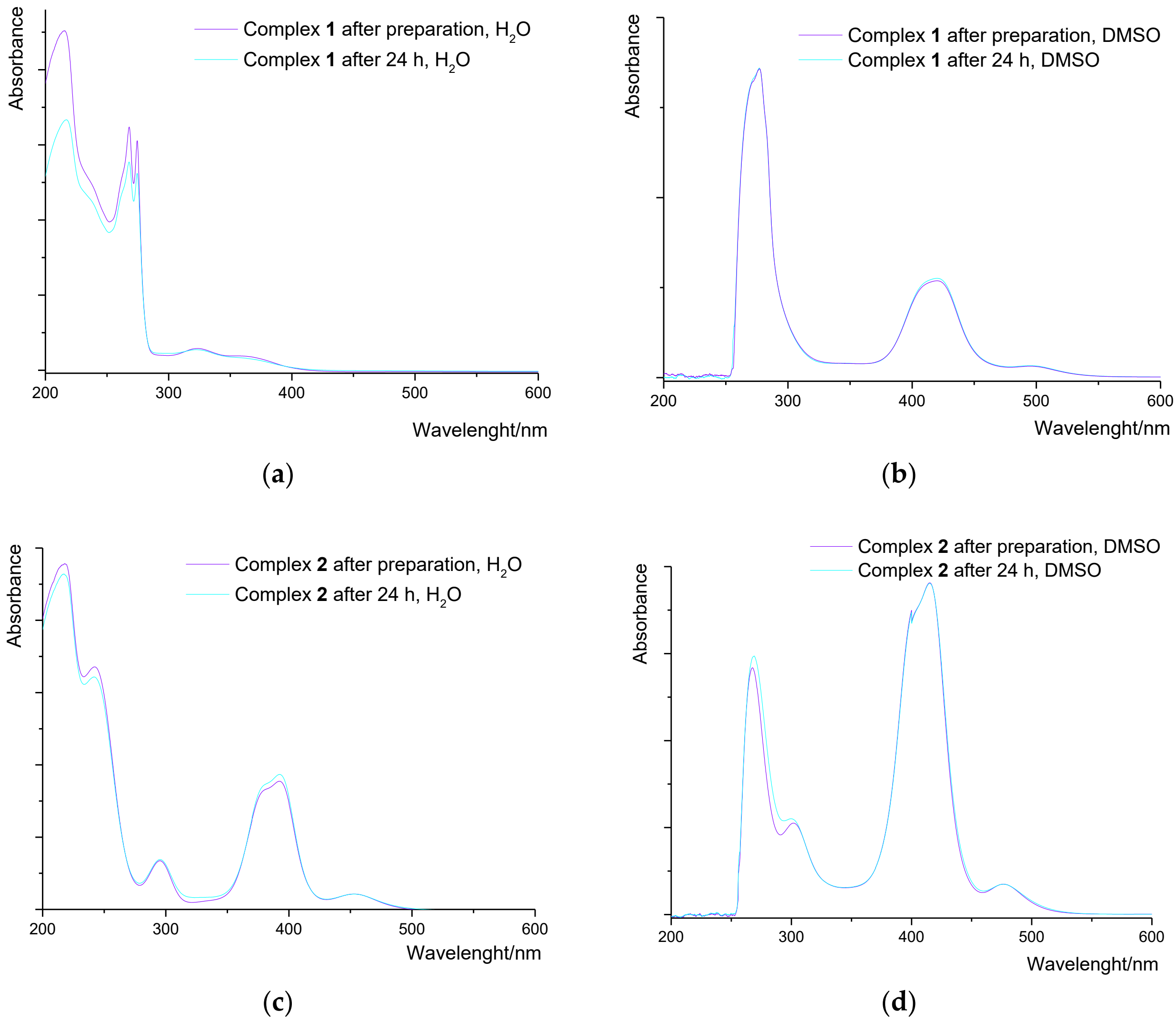

Figure S3. UV–Vis spectra of complexes **1** and **2** in aqueous solution (**a**, **c**) and in dimethyl sulfoxide solution (**b**, **d**), measured immediately after preparation and after 24 hours.

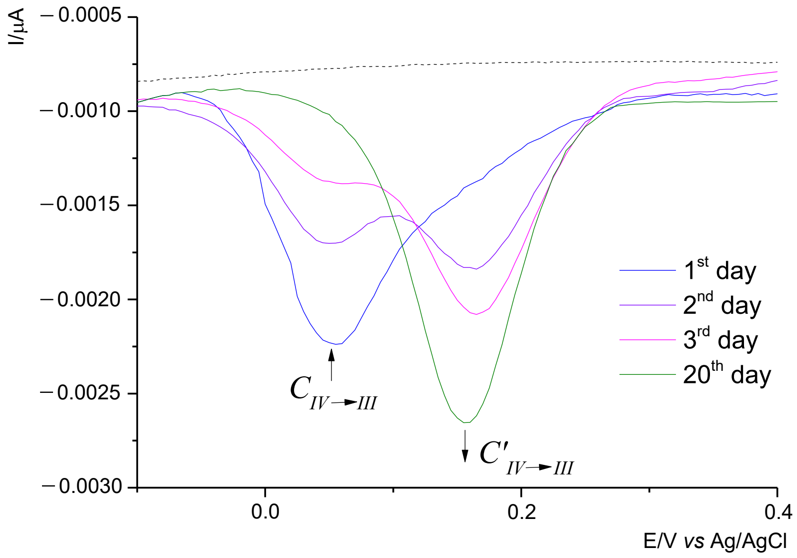

Figure S4. DPV curves for complex **1** recorded over time in the potential range from −0.1 to 0.4 V.

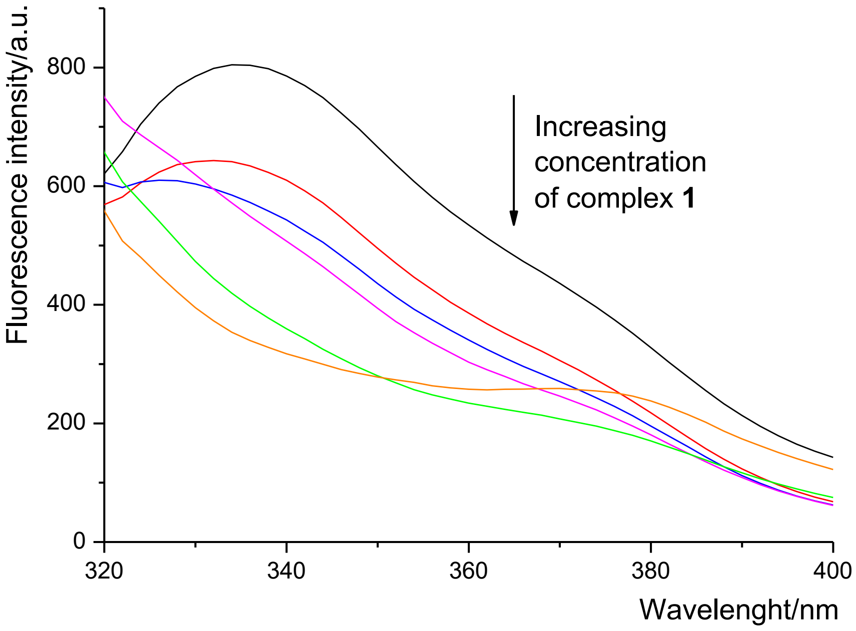

(**a**)

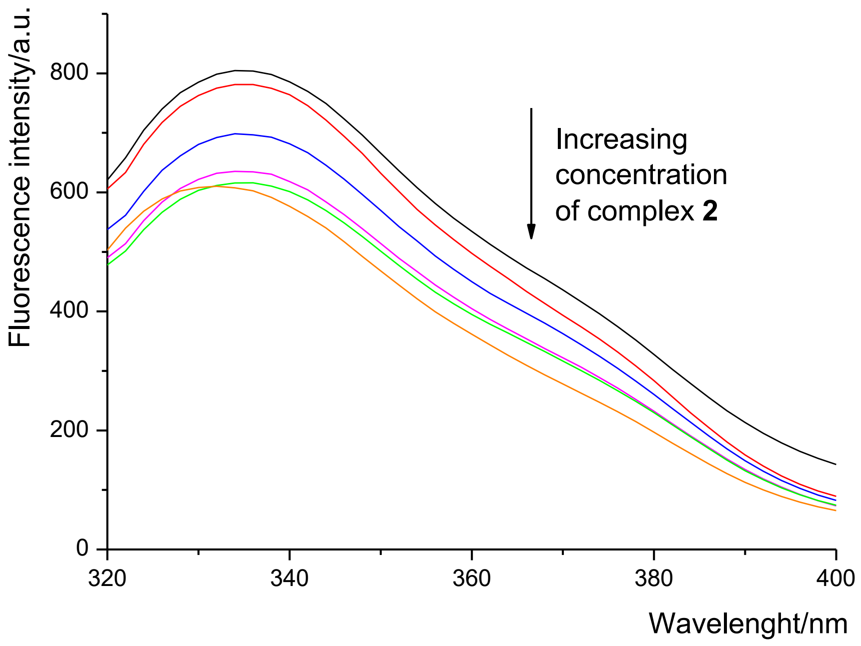

(**b**)

Figure S5. The fluorescence spectra of HSA in the absence or presence of complexes **1** (**a**) and **2** (**b**) at a temperature of 310 K. The concentration of HSA and the complexes were: 10 μM and 15.6–250 μM, respectively.

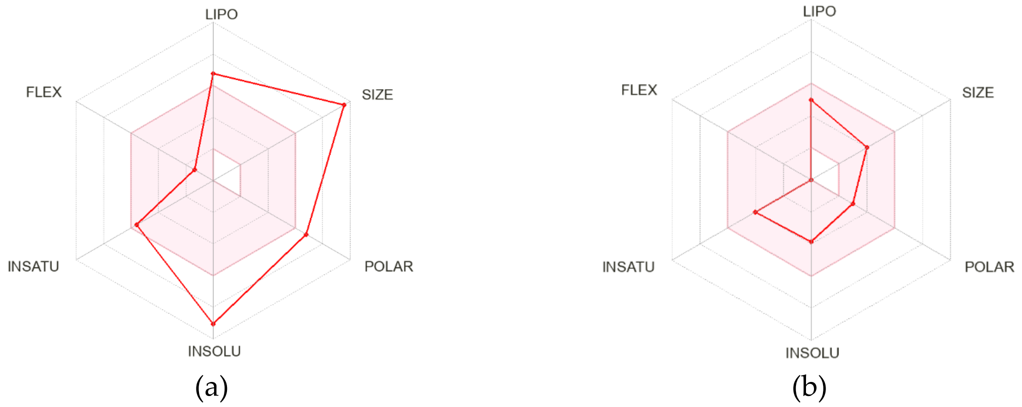

Figure S6. Bioavailability radar for complex **1** (**a**) and **2** (**b**).

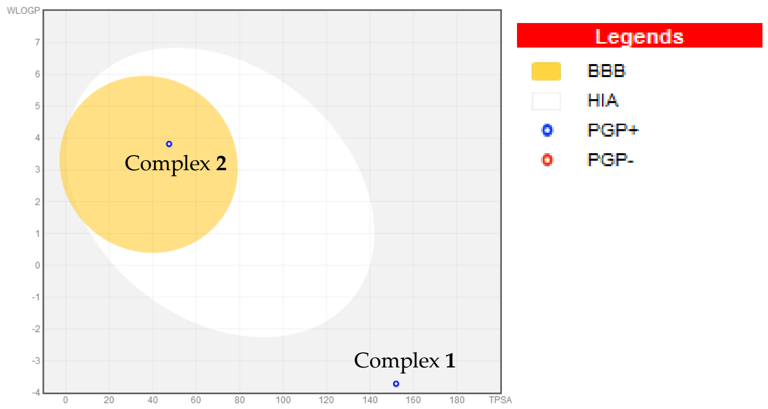

Figure S7. Boiled-Egg diagram for Ru compounds **1** and **2** illustrating lipophilicity (WLOGP) and polarity (TPSA). The white region represents human intestinal absorption, while the yellow area indicates permeation through the blood-brain barrier (generated by the SwissADME server accessed on 12 December 2024).
